# The Dynamics of Energy Dissipation and Xanthophyll Conversion in Arabidopsis Indicate an Indirect Photoprotective Role of Zeaxanthin in Slowly Inducible and Relaxing Components of Non-photochemical Quenching of Excitation Energy

**DOI:** 10.3389/fpls.2017.02094

**Published:** 2017-12-08

**Authors:** Eugen Kress, Peter Jahns

**Affiliations:** Plant Biochemistry, Heinrich-Heine-University Düsseldorf, Düsseldorf, Germany

**Keywords:** energy dissipation, non-photochemical quenching, photoinhibition, photosynthesis, xanthophyll cycle, zeaxanthin

## Abstract

The dynamics of non-photochemical quenching (NPQ) of chlorophyll fluorescence and the dynamics of xanthophyll conversion under different actinic light conditions were studied in intact leaves of *Arabidopsis thaliana*. NPQ induction was investigated during up to 180 min illumination at 450, 900, and 1,800 μmol photons m^−2^ s^−1^ (μE) and NPQ relaxation after 5, 30, 90, or 180 min of pre-illumination at the same light intensities. The comparison of wild-type plants with mutants affected either in xanthophyll conversion (*npq1* and *npq2*) or PsbS expression (*npq4* and *L17*) or lumen acidification (*pgr1*) indicated that NPQ states with similar, but not identical characteristics are induced at longer time range (15–60 min) in wild-type and mutant plants. In genotypes with an active xanthophyll conversion, the dynamics of two slowly (10–60 min) inducible and relaxing NPQ components were found to be kinetically correlated with zeaxanthin formation and epoxidation, respectively. However, the extent of NPQ was independent of the amount of zeaxanthin, since higher NPQ values were inducible with increasing actinic light intensities without pronounced changes in the zeaxanthin amount. These data support an indirect role of zeaxanthin in pH-independent NPQ states rather than a specific direct function of zeaxanthin as quencher in long-lasting NPQ processes. Such an indirect function might be related to an allosteric regulation of NPQ processes by zeaxanthin (e.g., through interaction of zeaxanthin at the surface of proteins) or a general photoprotective function of zeaxanthin in the lipid phase of the membrane (e.g., by modulation of the membrane fluidity or by acting as antioxidant). The found concomitant down-regulation of zeaxanthin epoxidation and recovery of photosystem II activity ensures that zeaxanthin is retained in the thylakoid membrane as long as photosystem II activity is inhibited or down-regulated. This regulation supports the view that zeaxanthin can be considered as a kind of light stress memory in chloroplasts, allowing a rapid reactivation of photoprotective NPQ processes in case of recurrent light stress periods.

## Introduction

Sunlight is not only the ultimate energy source for photosynthesis, but also the major source for the formation of reactive oxygen species (ROS) in chloroplasts. The latter is related to the fact that plants frequently absorb more light than they can use in photosynthesis. To cope with the damaging potential of excess light energy, plants have developed a number of strategies (i) to reduce the absorption of light (e.g., chloroplast movement, reduction of the antenna size), (ii) to reduce the amount of ROS production (e.g., alternative electron pathways, heat dissipation), or (iii) to detoxify produced ROS by antioxidants and antioxidative enzymes (Li et al., 2009). In the field, light intensities may vary in orders of magnitude at short time scale (seconds to minutes), which determines the demand for a flexible photoprotective response in the short-term. The dissipation of excess light as heat, also known as non-photochemical quenching (NPQ) of excitation energy, matches all requirements of such a flexible response to excess light. The overall NPQ is complex and comprises at least four different components, which have been termed (1) qE, the pH-regulated energy dissipation in the antenna of photosystem II (PSII) (Krause et al., 1982), (2) qT, state transitions (Allen et al., 1981), (3) qZ, zeaxanthin-dependent quenching (Nilkens et al., 2010), and (4) qI, photoinhibition (Krause, 1988). The relative contribution of the different components, which act on different time scales, to the total NPQ is variable and primarily depends, at least for plants grown under controlled lab conditions, on the illumination intensity and time.

### Characteristics of NPQ components

The qE component is the most rapidly (1–3 min) inducible and relaxing component, which is essentially controlled by the thylakoid lumen pH. Drop of the lumen pH below about pH 6.0 activates the PsbS protein (Li et al., 2000, 2002) which is supposed to induce conformational changes in the light-harvesting antenna of PSII (LHCII) through direct interaction with LHCII proteins (Correa-Galvis et al., 2016; Sacharz et al., 2017). qE is further modulated by the xanthophyll zeaxanthin (Zx) which is synthesized from violaxanthin (Vx) in the de-epoxidation reactions of the xanthophyll cycle (Jahns et al., 2009). Like qE activation through PsbS, also the formation of Zx is controlled by the lumen pH, which occurs at pH values <6 by activation of the lumen-localized Vx de-epoxidase (Hager, 1969). This pH regulation of qE allows a flexible adjustment of energy dissipation in response to fluctuating light intensities, and ensures that the dissipation of absorbed light energy is not active under light-limiting conditions, but only upon light-saturation of photosynthetic electron transport, as indicated by the lumen pH. The importance of the rapid down-regulation of qE after a transition from high light to low light, as given under fluctuating light, has been proven recently for plants with altered qE relaxation properties (Armbruster et al., 2014; Kromdijk et al., 2016).

The qT component of NPQ operates in the time scale of 10–20 min and serves the optimal balancing of the energy distribution between PSII and PSI (Allen, 2003). Activation of qT is triggered by the reduction of the plastoquinone pool through activation of the kinase STT7 (*Chlamydomonas*) or STN7 (*Arabidopsis*) (Bellafiore et al., 2005; Bonardi et al., 2005), which phosphorylates LHCII proteins. Phosphorylated LHCII is thought to detach from PSII and to associate with PSI, which decreases the functional antenna size of PSII and increases that of PSI. This process is reversible upon dephosphorylation of LHCII by the phosphatase TAP38/PPH1 (Pribil et al., 2010; Shapiguzov et al., 2010), which is particularly activated at high light. In land plants, the contribution of qT to the total NPQ is generally low and no significant contribution at all has been observed under saturating light conditions in Arabidopsis (Nilkens et al., 2010).

The qZ component of NPQ operates on a time scale from 10 to 30 min and has been assigned to Zx on basis of its kinetic correlation with the reconversion of Zx to Vx (Nilkens et al., 2010). In contrast to qE, qZ remains active in absence of a transthylakoid proton gradient (Dall'Osto et al., 2005) and thus in darkness. This NPQ component is likely identical with the component, which was earlier ascribed to a fast relaxing component of photoinhibition (Leitsch et al., 1994; Jahns and Miehe, 1996; Thiele et al., 1998).

The qI component of NPQ comprises all processes that are directly related to the photoinhibition of PSII. Photoinhibition of PSII (and thus activation of qI) typically occurs upon high light illumination at a time scale of hours, but maybe induced also at shorter time when photosynthesis is limited by unfavorable environmental conditions such as drought or low temperature. Photoinhibition of PSII is based on the damage of the D1 protein of the PSII reaction center (Aro et al., 1993). Recovery from photoinhibition requires the degradation of damaged D1 and its replacement by newly synthesized D1 protein. The PSII inhibition-repair cycle involves reversible phosphorylation of PSII reaction center proteins. In its phosphorylated state, damaged PSII monomerizes and moves from the grana region of the thylakoid membrane to stroma-exposed regions, where the repair of damaged D1 is supposed to take place (Tikkanen and Aro, 2012). Under most conditions, the degradation of damaged D1 protein is fully compensated by repair processes (Kyle et al., 1984), so that a net degradation of D1 occurs only under extreme light stress conditions or when the synthesis of D1 is blocked by inhibitors of protein synthesis in chloroplasts (Samuelsson et al., 1985). Hence, long-term exposure of plants to high light intensities involves the permanent repair of PSII and thus requires a highly flexible reorganization of the thylakoid membrane.

### Role of Zx in energy dissipation processes

The light-dependent and reversible conversion of Vx to Zx in plants has been discovered more than 50 years ago (Sapozhnikov et al., 1957; Yamamoto et al., 1962). This so-called violaxanthin cycle is known to be active in all land plants, brown algae and most of the green algae, while the diadinoxanthin cycle, which involves the reversible conversion of diadinoxanthin (Ddx) to diatoxanthin (Dtx) is active in a wide range of different algae including diatoms and haptophytes (for reviews see (Jahns et al., 2009; Goss and Jakob, 2010). The wide distribution of light-dependent xanthophyll conversion concomitant with the activation of NPQ supports an important photoprotective function of xanthophylls. This view is further supported by the fact, that also in photosynthetic organisms, which do not possess an active xanthophyll cycle, such as cyanobacteria, xanthophylls are supposed to serve central functions in energy dissipation (Wilson et al., 2006; Leverenz et al., 2015). It is a matter of a long lasting debate, however, whether the photoprotective function of xanthophylls is based on a direct function as quencher of excitation energy or an indirect function as modulator or regulator of energy dissipation, or as antioxidant in the lipid phase. These different modes of action depend on the respective localization of Zx. A direct function of Zx as quencher requires the binding of Zx at specific xanthophyll binding sites in antenna proteins. An indirect function of Zx likely involves an allosteric regulation of NPQ (Horton et al., 2000), which might be induced by either the release of Vx from xanthophyll binding sites (Kana et al., 2016) or the exchange of Vx by Zx at a xanthophyll binding site (Ruban and Horton, 1999; Kana et al., 2016), or through interaction of Zx with antenna proteins at the lipid-protein interface (Xu et al., 2015). Finally, a more general photoprotective function of Zx has been proposed for Zx in the lipid phase of the thylakoid membrane, either as modulator of membrane properties (Havaux, 1998) or as antioxidant (Havaux et al., 2004).

In the case of Zx, a function in energy dissipation has been proposed some 30 years ago (Demmig et al., 1987; Demmig-Adams, 1990; Demmig-Adams and Adams, 1996). This hypothesis was finally proven by the identification and characterization of Arabidopsis xanthophyll cycle mutants *npq1* and *npq2*, which are defective in the xanthophyll cycle enzymes Vx de-epoxidase (VDE) and Zx epoxidase (ZEP), respectively (Niyogi et al., 1998). The Zx-deficient *npq1* mutant exhibits a markedly reduced qE capacity, although a significant part of qE is still inducible in this mutant (Nilkens et al., 2010). In contrast, the Zx accumulating *npq2* mutant is particularly characterized by a more rapidly inducible and more slowly relaxing qE (Niyogi et al., 1998; Dall'Osto et al., 2005; Nilkens et al., 2010). It is still unclear whether Zx acts as a direct quencher of excitation energy or whether Zx functions in an indirect manner as modulator of energy dissipation (in case of qE) or simply as photoprotective component in the lipid phase of the membrane (in case of long-lasting NPQ states, such as qZ and qI). Evidence for a direct energy quenching by Zx has been derived so far only from time-resolved transient absorption measurements performed with isolated thylakoid membranes (Holt et al., 2005) or isolated antenna complexes (Ahn et al., 2008; Avenson et al., 2008). Based on these analyses the formation of a Chl-Zx charge transfer complex has been proposed to occur in minor antenna complexes (Lhcb4-6), but not in the major trimeric antenna complexes (LHCII) of PSII. However, recent analysis of minor and major antenna complexes isolated from dark-adapted (no Zx) or pre-illuminated (with Zx) plants, could not provide evidence for binding of significant amounts of Zx to single complexes, nor for any differences in the quenching capacity of the complexes in absence and presence of Zx (Xu et al., 2015).

While possible quenching sites and mechanisms have been studied in detail to clarify the role of Zx in qE, nearly no information is available for the role of Zx in more slowly relaxing NPQ components (qZ and qI), which remain active after relaxation of the transthylakoid pH gradient. Evidence for a role of Zx in qZ and qI has mainly been derived from the correlation of the dynamics of the Zx content and the dynamics of NPQ states. In fact, the qZ component of NPQ has been identified by the close correlation of the medium phase of NPQ relaxation and Zx epoxidation (Nilkens et al., 2010). Moreover, sustained inactivation of PSII induced by long-lasting high light stress has been correlated with sustained retainment of Zx in numerous studies (Ebbert et al., 2001, 2005; Adams et al., 2002; Demmig-Adams et al., 2006, 2012). Though such approaches are limited by the missing information about the underlying quenching mechanism, they can provide important information about the possible role of Zx in quenching. Here, we extended the investigation of the correlation of Zx and NPQ dynamics by comparing both processes at different light intensities, which induce to different extent the NPQ components qE, qZ and qI in Arabidopsis leaves. The use of different mutants affected in PsbS levels (*npq4* and *L17*), the xanthophyll cycle (*npq1* and *npq2*) and lumen acidification (*pgr1*) allowed us to correlate particularly the dynamics of the two slowly inducible and relaxing components qZ and qI with the dynamics of xanthophyll conversion. We show that NPQ components with similar characteristics of qZ and qI are formed in all genotypes although the Zx content varied largely among the genotypes. However, in genotypes with an active xanthophyll cycle, the reconversion of Zx to Vx was found to be closely correlated with the relaxation of slowly relaxing NPQ states. Based on these results, we hypothesize that Zx has no direct quenching function in qZ and qI, but rather functions as indirect regulator or photoprotective molecule in NPQ states that remain active in absence of a low lumen pH after moderate or severe high light stress.

## Materials and methods

### Plant material and growth conditions

*Arabidopsis thaliana* wild-type (ecotype Col-0) plants and the mutant plants *npq4* (Li et al., 2000), *L17* (Li et al., 2002), *npq1* and *npq2* (Niyogi et al., 1998), and *pgr1* (Munekage et al., 2001) were grown on soil at a light intensity of 150 μmol photons m^−2^ s^−1^ and a constant temperature of 20°C under long-day conditions (14 h light/10 h dark). Leaves from 5 to 6 weeks-old plants were used for all experiments.

### Chlorophyll fluorescence measurements

Chl a fluorescence was measured with detached leaves at 20°C with a pulse-amplitude-modulated fluorometer (PAM 101, Walz, Effeltrich, Germany). Leaves were placed on wet filter paper in a self-built cuvette, which allowed the control of the leaf temperature and the supply with moistened air throughout the experiment. For all experiments, leaves were dark-adapted for at least 2 h before start of the experiment. For NPQ induction, leaves were illuminated with white light for up to 180 min at light intensities of 450, 900, or 1,800 μmol photons m^−2^ s^−1^. Relaxation of NPQ was determined during a subsequent dark phase for up to 180 min. For the determination of NPQ induction, saturating white light pulses 4,000 μmol photons m^−2^s^−1^, duration 800 ms were applied every 20 s during the first 100 s of illumination and every 100 s for the remaining illumination time. For the determination of NPQ relaxation, saturating light pulses were spaced 20 s for the first 100 s, followed by 8 flashes spaced 100 s and flashes spaced 500 s for the remaining time. Control measurements in absence of actinic light indicated that the saturating light pulses alone neither induced reasonable NPQ (>0.02) during the induction of NPQ, nor affected the NPQ relaxation kinetics. NPQ was determined as (Fm/Fm'-1) (Krause and Jahns, 2003).

### Correlation analyses

The correlation of NPQ and Zx was evaluated by linear regression analysis of plots of NPQ values vs. the Zx content. Regression analysis was performed with Grafit software (Grafit 5, Erithacus Software Limited, UK) and the resulting Pearson's correlation coefficients were determined.

### Pigment analysis

To determine the formation of Zx, detached leaves from dark-adapted plants were floated on water and illuminated at the same intensities of white light as used for NPQ induction for up to 180 min. Reconversion of Zx to Vx was induced by transfer of pre-illuminated leaves to darkness. At the indicated times, leaves were frozen in liquid N_2_ and stored at −80°C. Pigments were extracted with acetone and quantified by reverse phase HPLC as described earlier (Färber et al., 1997).

## Results

We determined the dynamics of NPQ induction and relaxation in six genotypes and at three different actinic light (AL) intensities of 450, 900, and 1,800 μmol photons m^−2^ s^−1^ in comparison with the dynamics of Zx synthesis and reconversion. The overall data are summarized as an overview in Figure S1 (NPQ dynamics) and Figure S2 (Zx dynamics). For all experiments, dark-adapted leaf discs were illuminated for up to 180 min to determine NPQ induction and Zx synthesis, whereas dark relaxation of NPQ and Zx reconversion was measured for up to 180 min after four different times of pre-illumination (5, 30, 90, and 180 min). This experimental setup was chosen to determine in particular the characteristics of the slowly developing and relaxing NPQ components qZ and qI in comparison with Zx dynamics. The three AL intensities (for the sake of simplicity, the unit μmol photons m^−2^ s^−1^ will be abbreviated as μE throughout the text and all figures) represent illumination conditions, which are expected to either induce predominantly the qE component only (450 μE) or both the qE and qZ components (900 μE) or additionally pronounced photoinhibitory qI quenching (1,800 μE). Wild-type (WT) plants were analyzed in comparison with mutants affected in the major regulators of NPQ, namely the lumen pH (*pgr1*), the xanthophyll cycle (*npq1* and *npq2*), and the PsbS protein (*npq4* and *L17*). In general, the data supported the well-known differences among the different genotypes. Pronounced rapid (within few min) pH-dependent NPQ induction and relaxation was only detectable in WT, *L17*, and *npq2* plants, while the absence or strong reduction of rapid NPQ changes in the other genotypes is related to limited lumen acidification (*pgr1*), the absence of PsbS (*npq4*), or the absence of Zx (*npq1*) (Figure S1). Zx formation was detectable with similar characteristics in all genotypes with an active xanthophyll cycle, except for *pgr1* plants, which showed a delayed and reduced Zx synthesis (Figure S1), reflecting the limited lumen acidification in this mutant. In the following, the characteristics of the NPQ dynamics are presented and explained in more detail, and further compared with the Zx dynamics. The pH-regulated qE component is denoted as rapidly inducible/relaxing NPQ component, while the remaining NPQ processes are termed as slowly inducible/relaxing NPQ components, comprising qZ and qI. It should be noted, that the qT component of NPQ, which is related to state transitions, is not included in the slow NPQ components, because former analysis of the qT-deficient *stn7* mutant has shown that qT does not contribute significantly to NPQ in Arabidopsis under our experimental conditions (Nilkens et al., 2010).

### NPQ induction at different AL intensities

**Figure 1:**
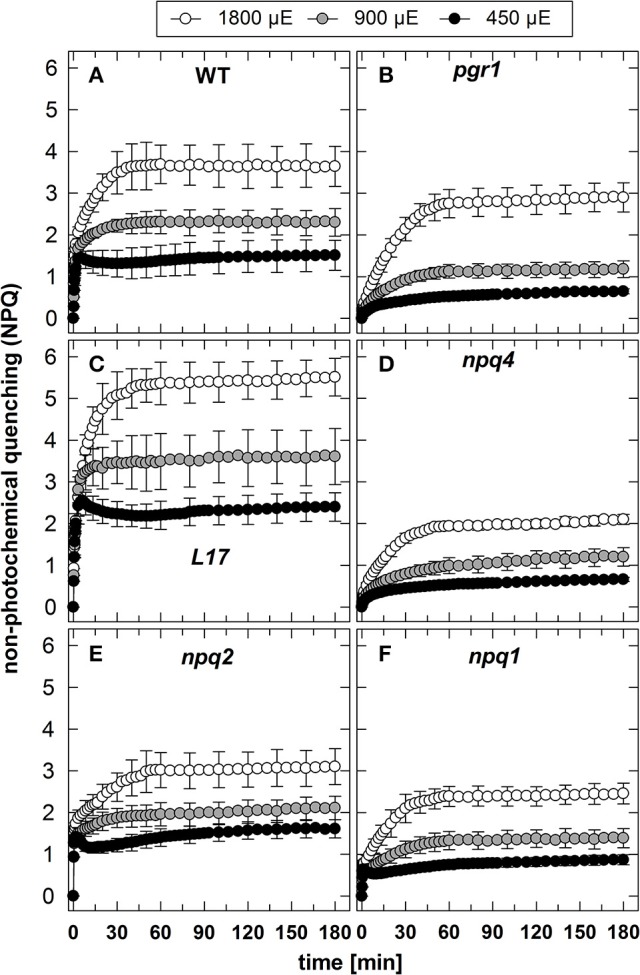
NPQ induction. The induction of NPQ during 180 min of illumination at three different actinic light intensities (450, 900, and 1,800 μE of white light) was determined for **(A)** WT, **(B)** *pgr1*, **(C)** *L17*, **(D)** *npq4*, **(E)**, *npq2*, and **(F)** *npq1* plants. During the whole measurements, detached leaves were placed on wet paper in a temperature-controlled cuvette (20°C) under permanent supply with ambient air. Mean values ± SE of 3–6 independent measurements are shown.

The NPQ induction at the three different AL intensities is illustrated in Figure 1. It is obvious that the genotypes differ predominantly in the induction of rapid NPQ processes, which reflect the pH-regulated qE component of NPQ. As expected, no rapid NPQ induction was detectable at all AL intensities in *pgr1* (Figure 1B) and *npq4* (Figure 1D) plants, underlining the known essential role of the lumen pH (Briantais et al., 1979) and PsbS (Li et al., 2000), respectively, for qE activation. The partial reduction of qE in the *npq1* (Figure 1F) mutant supports the idea that Zx modulates the qE capacity, but is not an essential prerequisite for qE (Horton et al., 2000; Nilkens et al., 2010). Comparing the impact of the different AL intensities on qE induction in the three genotypes with high qE capacity (WT, *L17, npq2*) it is apparent that almost the maximum qE capacity is induced at the lowest intensity of 450 μE (Figures 1A,C,E). This indicates that the lumen pH induced at the lowest AL intensity is already low enough to nearly saturate qE induction. Consequently, the increase of the AL intensity to 900 and 1,800 μE predominantly leads to the additional induction of the slowly activated NPQ components qZ and qI (Figure 1).

**Figure 2:**
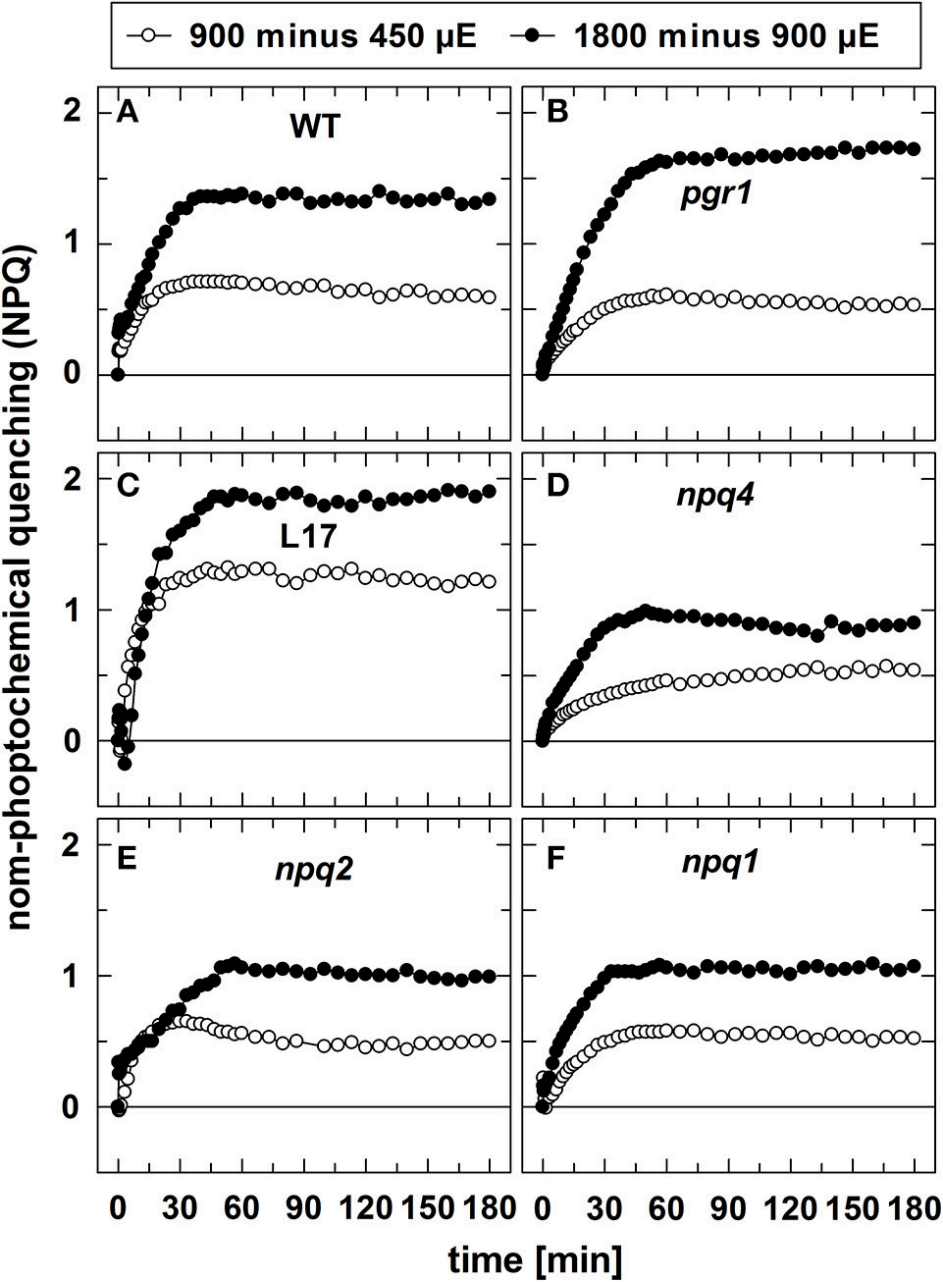
Differences in NPQ induction upon increase of light intensities. To visualize the differences in NPQ induction upon increase of the actinic light intensity from either 450 to 900 μE or from 900 to 1,800 μE, the differences of the respective curves shown in in Figure 1 were plotted for all genotypes. **(A)** WT, **(B)** *pgr1*, **(C)** *L17*, **(D)** *npq4*, **(E)**, *npq2*, and **(F)** *npq1* plants.

In contrast to qE, however, these two slowly inducible NPQ components were generated to a similar extent in all genotypes, except for the *L17* mutant (Figure 1C), which revealed a more pronounced increase of NPQ than all other genotypes. For a better evaluation of the increase of NPQ induced by increasing AL intensities, we plotted for each genotype the differences of the NPQ induction curves determined at the different AL intensities (900 minus 450 μE or 1,800 minus 900 μE; Figure 2). The increase of the AL intensity from 450 to 900 μE induced an increase of the maximum NPQ level by about 1.2 in *L17* plants (Figure 2C), but only by about 0.5 in all other genotypes (Figure 2). In plants with high qE capacity (WT, *L17* and *npq2*), the increased NPQ capacity developed faster (half-rise time about 5 min) than in the other genotypes (half-rise time about 15 min). This phase has earlier been assigned to the activation of qZ quenching, but the presence of this component in *npq2* and *npq1* plants indicates that a similar NPQ component is inducible independent of Zx. The increase of the AL intensity from 900 to 1,800 μE induced a further increase of the maximum NPQ level in all genotypes. This increase can be predominantly assigned to the qI component of NPQ and occurred with similar kinetics in all genotypes but with different amplitudes in the range from about 1.0 (*npq1, npq2*, and *npq4*) to about 1.5–2.0 (WT, *pgr1*, and *L17*) (see Figure 2). Hence, the induction of the slowly developing NPQ components, qZ and qI, showed quite similar characteristics in all genotypes, and is thus independent of the lumen pH, the PsbS protein and Zx synthesis.

### Zx synthesis at different AL intensities

**Figure 3:**
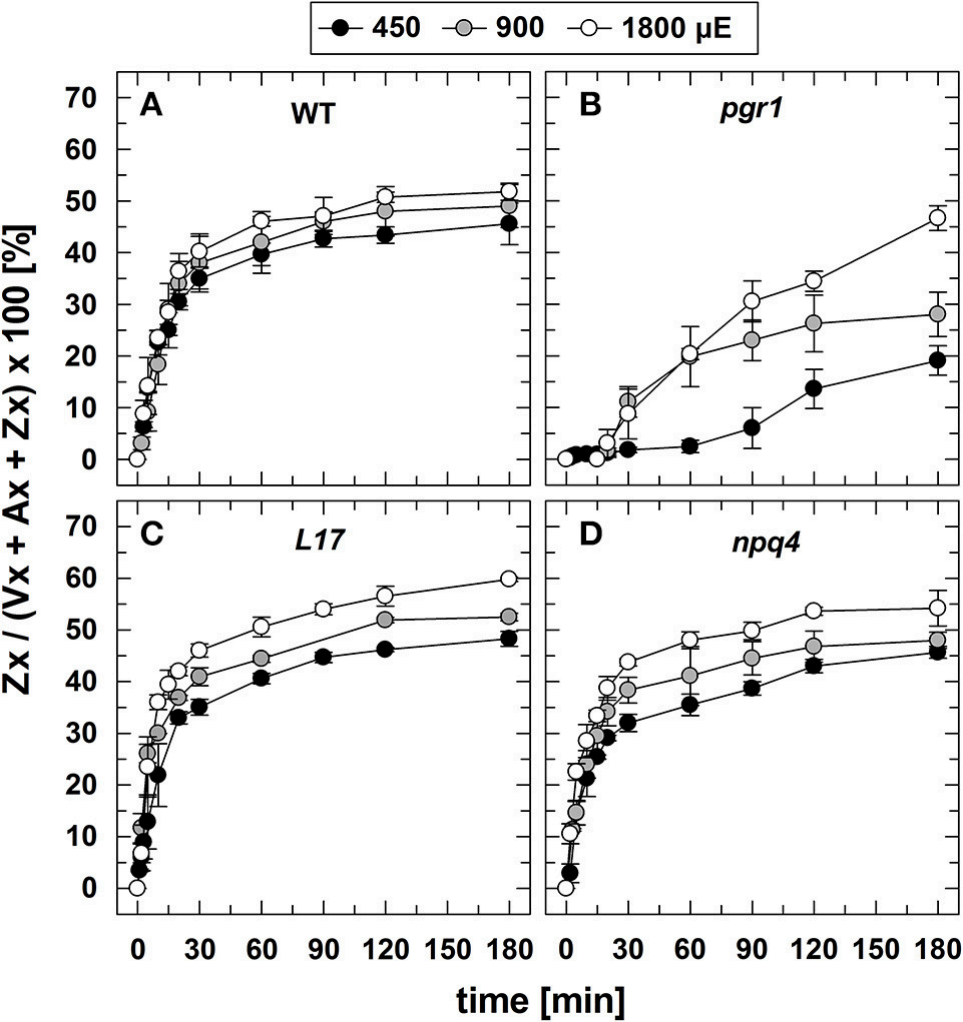
Zx synthesis. The synthesis of Zx during 180 min of illumination at three different actinic light intensities (450, 900, and 1,800 μE of white light) is shown for **(A)** WT, **(B)** *pgr1*, **(C)** *L17*, **(D)** *npq4* plants. Detached leaves were placed on water in temperature-controlled cuvette (20°C). At indicated time, leaves were rapidly frozen in liquid N_2_ and the pigment composition was determined by HPLC analyses. The Zx content in % of the total VAZ-pool size (= sum of Vx, Ax and Zx) is shown. Data represent mean values ± SE of 3–4 independent measurements.

The conversion of Vx to Zx during illumination (Figure 3) was determined under the same experimental conditions, aside from the fact that no saturating flashes were applied. Since no changes in the Zx content was detectable for the two xanthophyll cycle mutants *npq1* (0% Zx) and *npq2* (100% Zx), no data are shown for these two genotypes. For WT (Figure 3A), *L17* (Figure 3C), and *npq4* (Figure 3D) plants, both the extent and the kinetics of Zx synthesis were very similar at all AL intensities, indicating that Vx de-epoxidation characteristics are independent of the PsbS protein. Moreover, the maximum Zx content was only slightly increased upon increase of the AL intensity from 450 to 900 and 1,800 μE. This suggests that the light-induced lumen acidification is close to saturation of the VDE activity already at the lowest AL intensity, in accordance with the observed characteristics of qE (Figure 2). Compared to these genotypes, however, the formation of Zx was strongly delayed and reduced in *pgr1* mutants (Figure 3B), which reflects the limited lumen acidification reported for this mutant (Jahns et al., 2002).

### Kinetic correlation of Zx synthesis and NPQ induction

**Figure 4:**
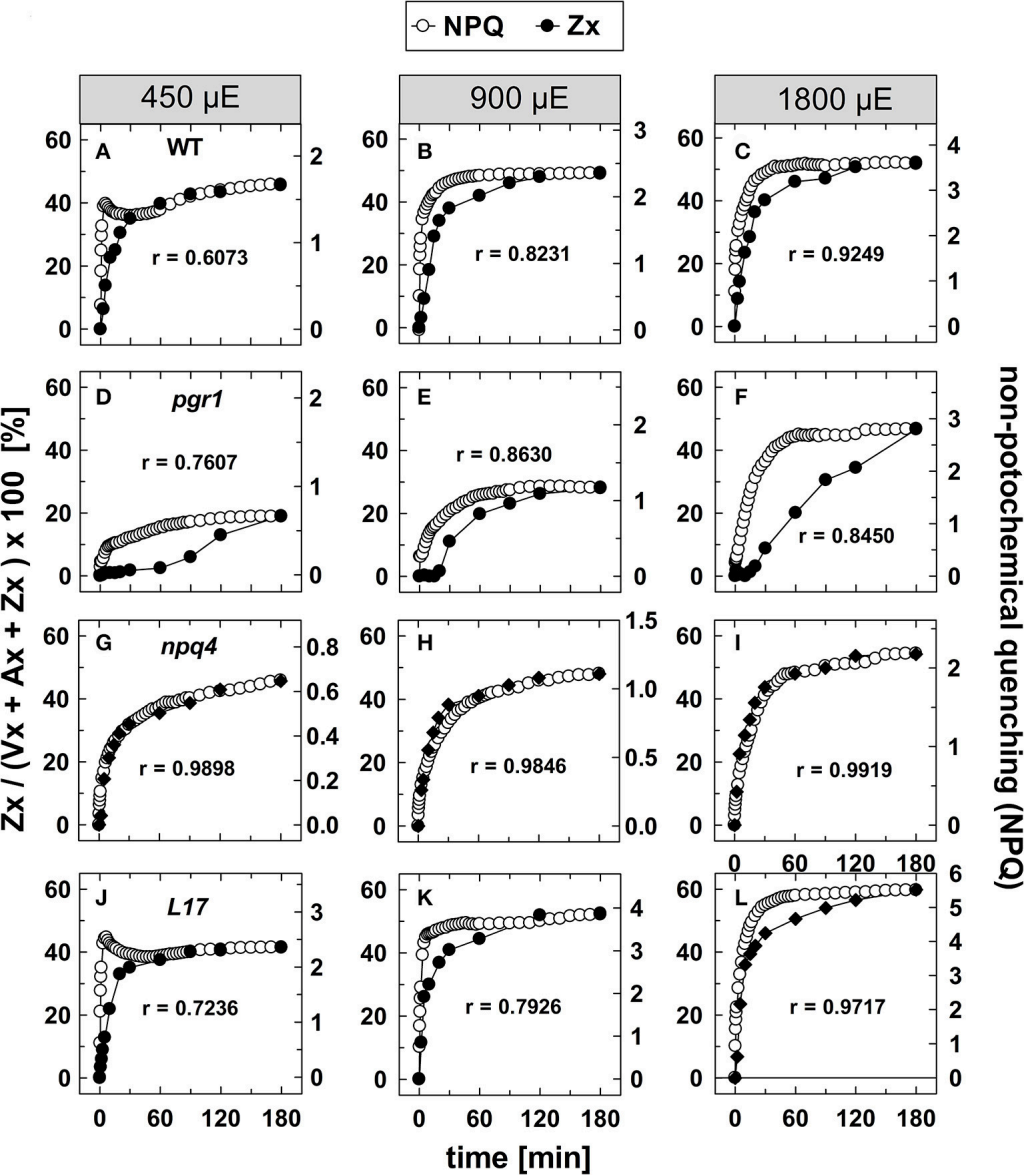
Comparison of NPQ induction and Zx synthesis. The time course of NPQ induction and Zx synthesis is compared for the four genotypes with an active xanthophyll cycle for the three actinic light intensities of 450, 900, and 1,800 μE: **(A–C)** WT, **(D–F)** *pgr1*, **(G–I)** *npq4*, and **(J–L)** *L17*. The data were taken from Figure 1 (NPQ) and Figure 3 (Zx). The determined Pearson's correlation coefficient r is indicated in each panel.

To evaluate the kinetic correlation of NPQ induction and Zx synthesis, both parameters were plotted in the same diagram, and the maximal amplitude was scaled to the same size (Figure 4). From plots of NPQ vs. Zx (exemplarily shown in Figure S3), the respective Pearson's correlation coefficient r was calculated, as indicated in each panel. For the two genotypes with an active qE quenching (WT and *L17*), the induction of NPQ was found to be clearly faster than Zx synthesis at the two lowest AL intensities (Figures 4A–C,J–L, respectively), resulting in rather low correlation coefficients over the entire time range. At the lowest AL intensity of 450 μE and in the time range from 30 to 180 min illumination, however, the slow increase of NPQ showed very similar kinetics than Zx synthesis in both genotypes (Figures 4A,J). At the two higher AL intensities, the correlation coefficient increased, indicating that with increasing contribution of the slowly inducible NPQ components (qZ and qI) to the total NPQ, the kinetic correlation of NPQ induction and Zx synthesis is higher in these two genotypes (Figures 4B,C,K,L). In *pgr1* plants, Zx synthesis was much slower than NPQ induction throughout the whole illumination period at all AL intensities (Figures 4D–F), and thus yielded low correlation coefficients. Strikingly, NPQ induction and Zx synthesis were kinetically closely correlated in the full time range in *npq4* plants (Figures 4G–I) at all AL intensities as obvious from the high correlation coefficients (>0.98). These data indicate that Zx synthesis is kinetically correlated rather with the slowly developing NPQ states only.

**Figure 5:**
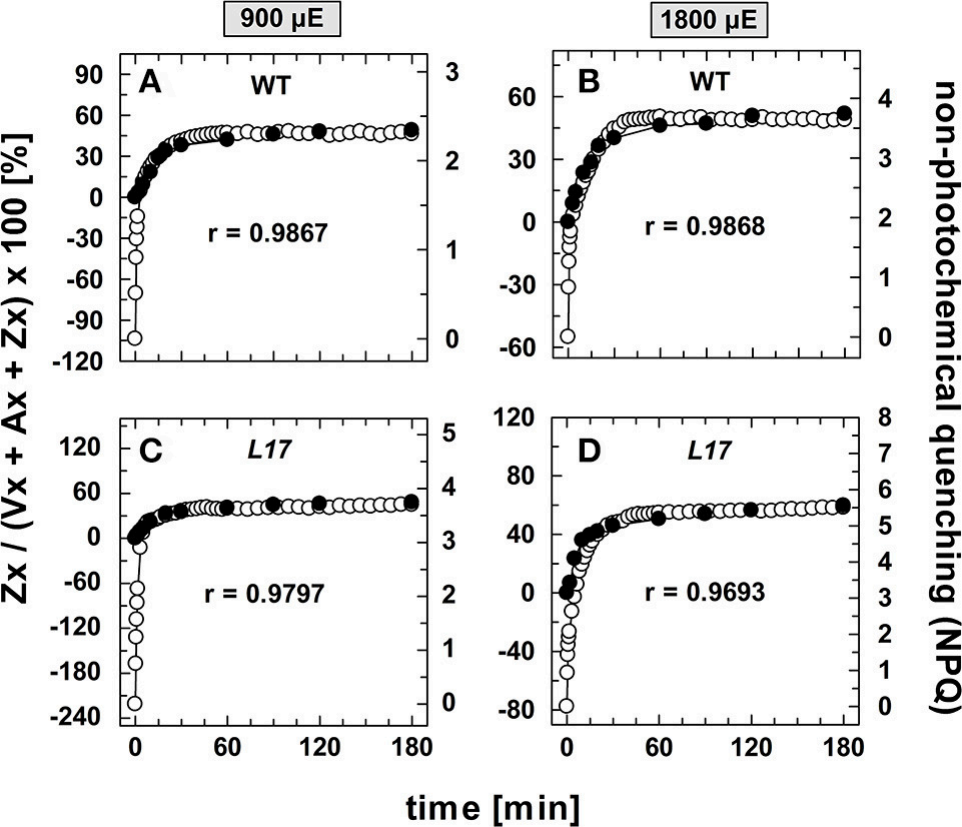
Comparison of the kinetics of NPQ induction and Zx synthesis in WT and *L17* plants. The data for NPQ induction (open circles) and Zx synthesis (filled circles) are compared for WT plants **(A,B)** and *L17* plants **(C,D)** at 900, **(A,C)** and 1,800 μE **(B,D)**. For direct comparison, the data for Zx synthesis were fitted to match the amplitudes of the slowly developing (> 2 min) NPQ components, only. The data were taken from Figure 1 (NPQ) and Figure 3 (Zx). The determined Pearson's correlation coefficient r is indicated in each panel.

The correlation of Zx synthesis with the induction of slowly developing NPQ states in the qE-active genotypes (WT and *L17*) at the two highest AL intensities is depicted more detailed in Figure 5. Here, the data were scaled in a way that the zero point of the scale for Zx synthesis matches the NPQ scale at the end point of the qE induction (about 1.5 in WT plants and 3.1 in *L17* plants). Hence, this plot emphasizes the correlation of Zx induction with the slowly inducible NPQ states is shown (compare also Figures S3B,D). This analysis yielded high correlation coefficients (>0.97) and clearly demonstrates that the kinetics of Zx synthesis is kinetically closely correlated with the induction of the slowly developing NPQ states. It should be noted, however, that the activation of these NPQ states does not necessarily require Zx, since NPQ states with similar characteristics were induced in absence of Zx (*npq1*) and in presence of 100% Zx (*npq2*).

### NPQ relaxation characteristics in dependence of the intensity and duration of pre-illumination

**Figure 6:**
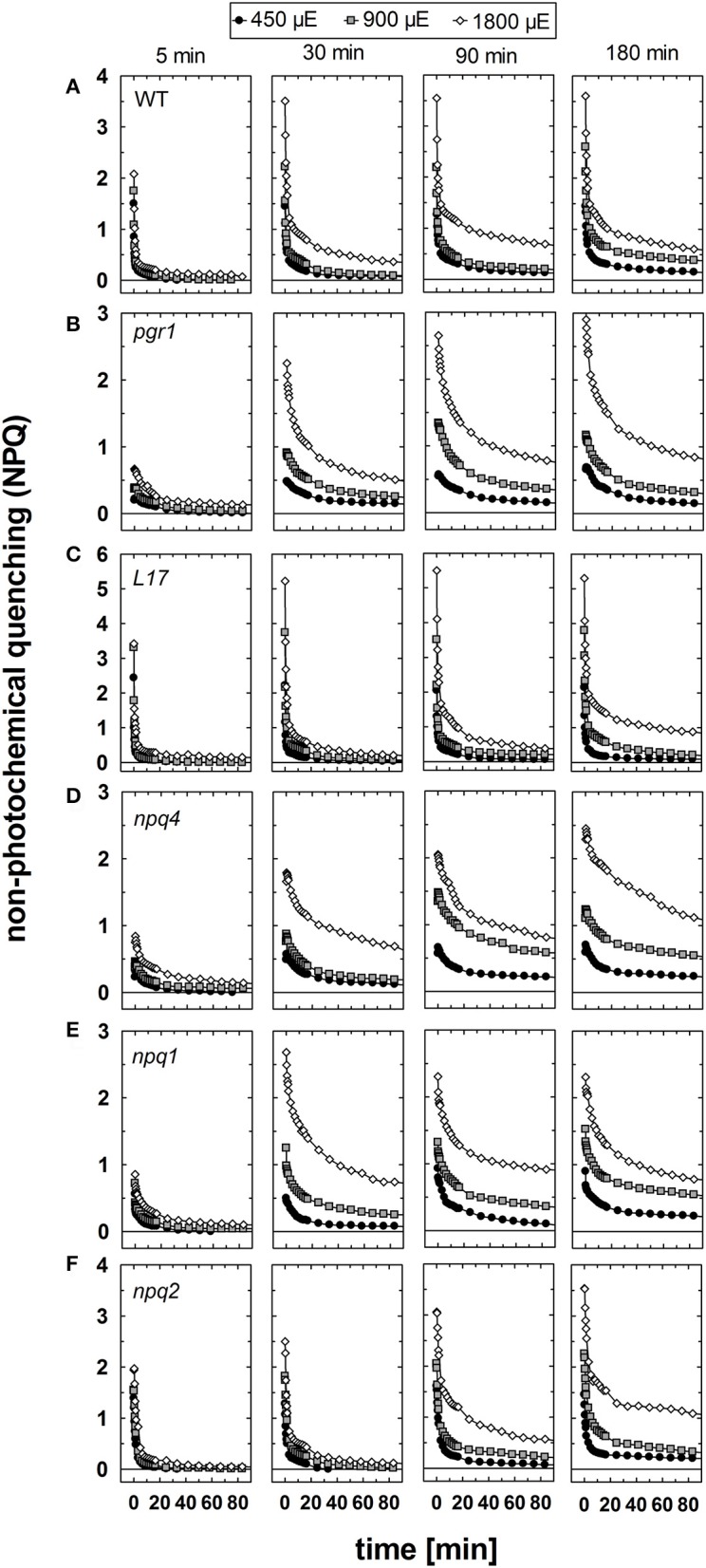
NPQ relaxation. The dark relaxation of NPQ after pre-illumination at three different actinic light intensities (450, 900, and 1,800 μE of white light) for 5, 30, 90, and 180 min was determined for **(A)** WT, **(B)** *pgr1*, **(C)** *L17*, **(D)** *npq4*, **(E)**, *npq2*, and **(F)** *npq1* plants. During the whole measurements, detached leaves were placed on wet paper in a temperature-controlled cuvette (20°C) under permanent supply with ambient air. Mean values of 3–6 independent measurements are shown. For clarity, error bars, which ranged from 0.1 to 0.3 in all cases, are not shown.

The relaxation of NPQ after different times of pre-illumination at the three AL intensities is summarized in Figure 6 and the detailed analysis of the NPQ relaxation kinetics characteristics is listed in Tables S1–S3 for the three AL intensities 450, 900, and 1,800 μE, respectively. In genotypes with an active qE mechanism (WT, *L17* and *npq2*), rapid (= within 3 min) relaxation of NPQ dominated under all conditions (Figures 6A,C,F and Tables S1–S3). The decay time (τ) of qE was in the range from 10 to 50 s for WT and *L17* plants, and in the range from 100 to 200 s for *npq2* plants (Tables S1–S3), in agreement with earlier work (Nilkens et al., 2010). A clear additional contribution of a more slowly relaxing component was detectable in these genotypes after increasing illumination times, particularly at the highest Al intensity of 1,800 μE. This component has been assigned to the qZ component of NPQ (Nilkens et al., 2010). and its relaxation time increased with increasing illumination time at all AL intensities from 200 to 500 s after 5 min pre-illumination up to 2,000–5,000 s after 180 min of pre-illumination (Tables S1–S3). It is important to note, that this component, which has been correlated with the epoxidation of Zx to Vx in WT plants, is present with similar characteristics also in *npq2* plants, which are unable to convert Zx to Vx. A third component, which was irreversible in the time range of the measurements and thus was assigned to the qI component (= photoinhibition) was detectable particularly at longer times of pre-illumination. The amplitude of this component increased with increasing illumination time and increasing AL intensities in all genotypes (Figure 6 and Tables S1–S3).

In *pgr1* (Figure 6B) and *npq4* (Figure 6D) mutants, which are impaired in qE activation, a rapidly relaxing NPQ component was missing. The most rapid component of NPQ relaxation in these two mutants matched in most cases the characteristics of the medium (qZ) component of NPQ relation observed in WT plants (Tables S1–S3). In contrast to the qZ phase in WT plants, however, this component became accelerated in *pgr1* and *npq4* after illumination with the highest AL intensity of 1,800 μE. Both mutants further showed a more slowly relaxing component, which was characterized by very slow kinetics (τ > 2,000 s) and additionally, like in WT plants, an irreversible part of NPQ, which can be assigned to qI.

Unique NPQ relaxation characteristics were found for *npq1* plants (Figure 6E). In this mutant, a rapidly relaxing component was detectable, though with lower amplitudes than in WT plants. The latter is related to a fraction of qE that is activated even in absence of Zx (Figure 1). It should be noted, however, that the amplitude of rapidly relaxing NPQ in *npq1* was reduced in comparison to the amplitude of the rapidly induced NPQ under all conditions (Tables S1–S3). Moreover, the relaxation kinetics were clearly retarded with increasing time of pre-illumination at all AL intensities. The slowly relaxing NPQ components, however, showed similar characteristics in comparison with WT plants. Hence, NPQ relaxation similar to the qZ component of WT plants is present even in absence of Zx.

In conclusion, NPQ relaxation in the different genotypes differed predominantly with respect to the rapidly relaxing qE component, but apart from that showed very similar responses to varying light treatments. Increasing illumination time led to retardation of NPQ relaxation in all genotypes and at all AL intensities. For all times of pre-illumination, this retardation was more apparent at higher AL intensities, reflecting the increased induction of photoinhibitory processes under these conditions. However, the retardation of NPQ relaxation was less pronounced in genotypes with an active qE mechanism, namely WT (Figure 6A)*, L17* (Figure 6C), and *npq2* (Figure 6F), in comparison to those with an impaired qE quenching, *pgr1* (Figure 6B), *npq4* (Figure 6D) and *npq1* (Figure 6E), indicating a photoprotective role of qE quenching. However, the maximum absolute levels of slowly relaxing NPQ components, i.e., the amplitude of qZ and qI, was similar in most genotypes, varying from about 1.5 to 2.0. Only *pgr1* mutant plants showed an increased level of slowly relaxing NPQ with an amplitude of about 2.5–3.0.

### Zx epoxidation characteristics

**Figure 7:**
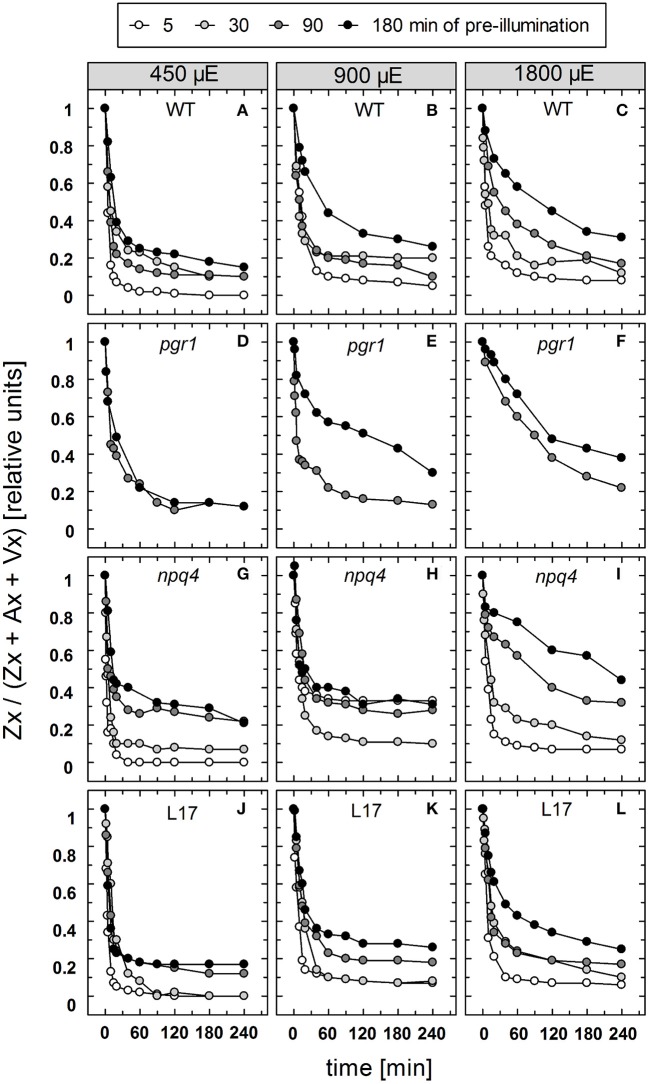
Zx epoxidation. The reconversion of Zx to Vx after pre-illumination at three different actinic light intensities (450, 900, and 1,800 μE of white light) for 5, 30, 90, and 180 min was determined for WT **(A–C)**, *pgr1* **(D–F)**, *npq4* **(G–I)**, and *L17* **(J–L)** plants. During the whole experiment, detached leaves were placed on water in temperature-controlled cuvette (20°C). At indicated time, leaves were rapidly frozen in liquid N_2_ and the pigment composition was determined by HPLC analyses. The Zx content in % of the total VAZ-pool size (= sum of Vx, Ax and Zx) is shown. Data represent mean values of 3–4 independent measurements. For clarity, error bars, which ranged from 1 to 5% in all cases, are not shown.

The reconversion of Zx to Vx, i.e., the epoxidation of Zx, was determined under the same conditions for the four genotypes with an active xanthophyll cycle (Figure S2). For a better comparison of the epoxidation kinetics, the data are depicted in a normalized way in Figure 7. For Zx epoxidation, no detailed kinetics analysis was performed for two reasons: First, due to the rather low amount of data points the amplitudes and kinetics could not be fitted with sufficient reliability. Second, Zx epoxidation does not follow exponential decay characteristics, but represents a two-stepped consecutive reaction, which further limits accurate fitting with few data points. The reconversion of Zx to Vx showed in general the same features as the NPQ relaxation, except for the absence of a rapid phase with decay times in the range from 1 to 2 min. However, similar to the two slower NPQ relaxation components, epoxidation of Zx was retarded in response to increasing illumination time and increasing AL intensity for all genotypes (Figure 7). For *pgr1* plants, epoxidation was only determined for the two longest times of pre-illumination (90 and 180 min), because only very low levels of Zx were formed at shorter illumination times of 15 and 30 min (Figure 3).

### Kinetic correlation of Zx epoxidation and NPQ relaxation

**Figure 8:**
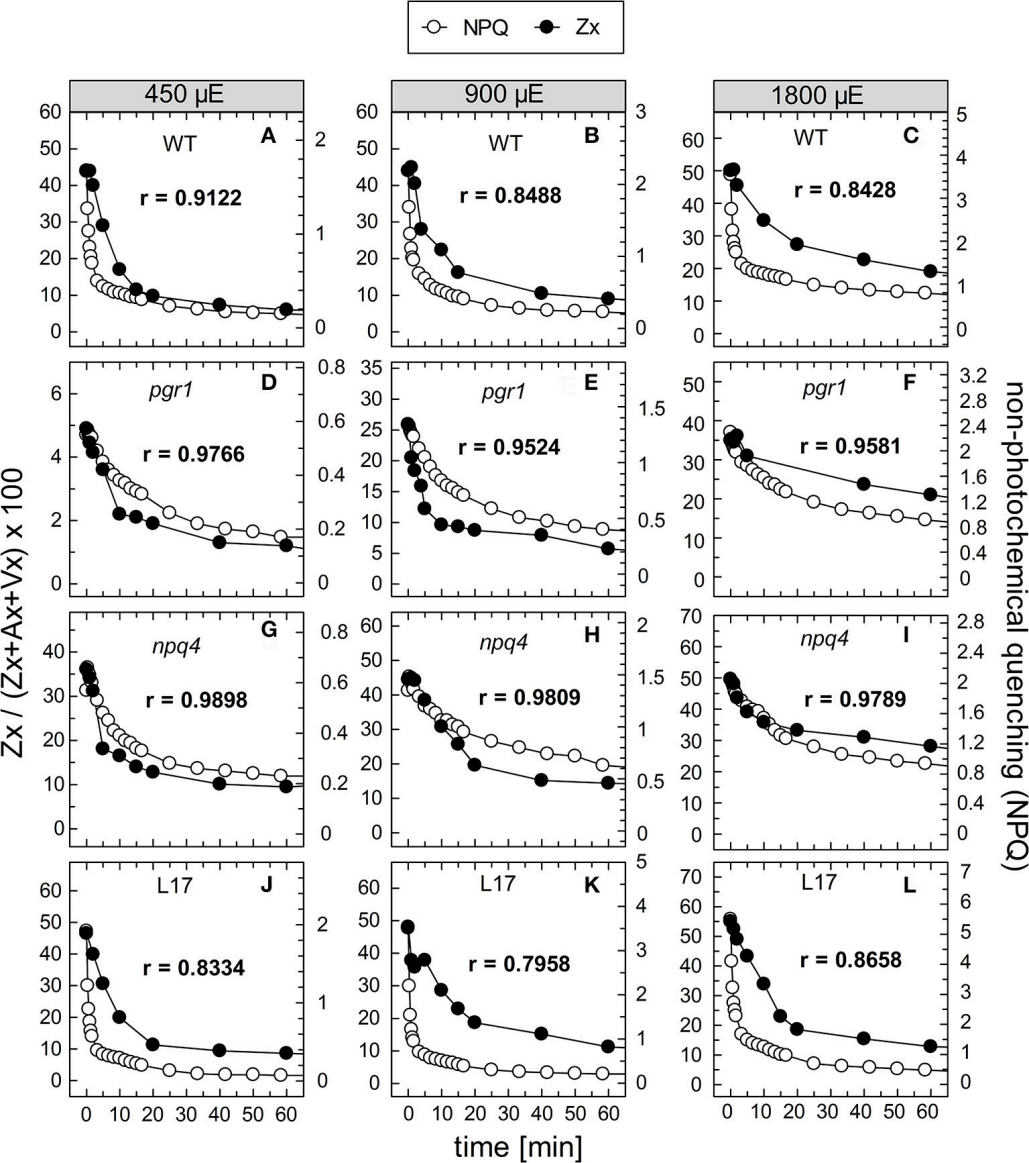
Comparison of NPQ relaxation and Zx epoxidation after 90 min of pre-illumination. The time course of NPQ relaxation and Zx epoxidation after 90 min of pre-illumination at the three actinic light intensities of 450, 900, and 1800 μE is compared for the four genotypes with an active xanthophyll cycle: **(A–C)** WT, **(D–F)** *pgr1*, **(G–I)** *npq4*, and **(J–L)** *L17*. The data were taken from Figure 6 (NPQ) and Figure 7 (Zx). The determined Pearson's correlation coefficient r is indicated in each panel.

**Figure 9:**
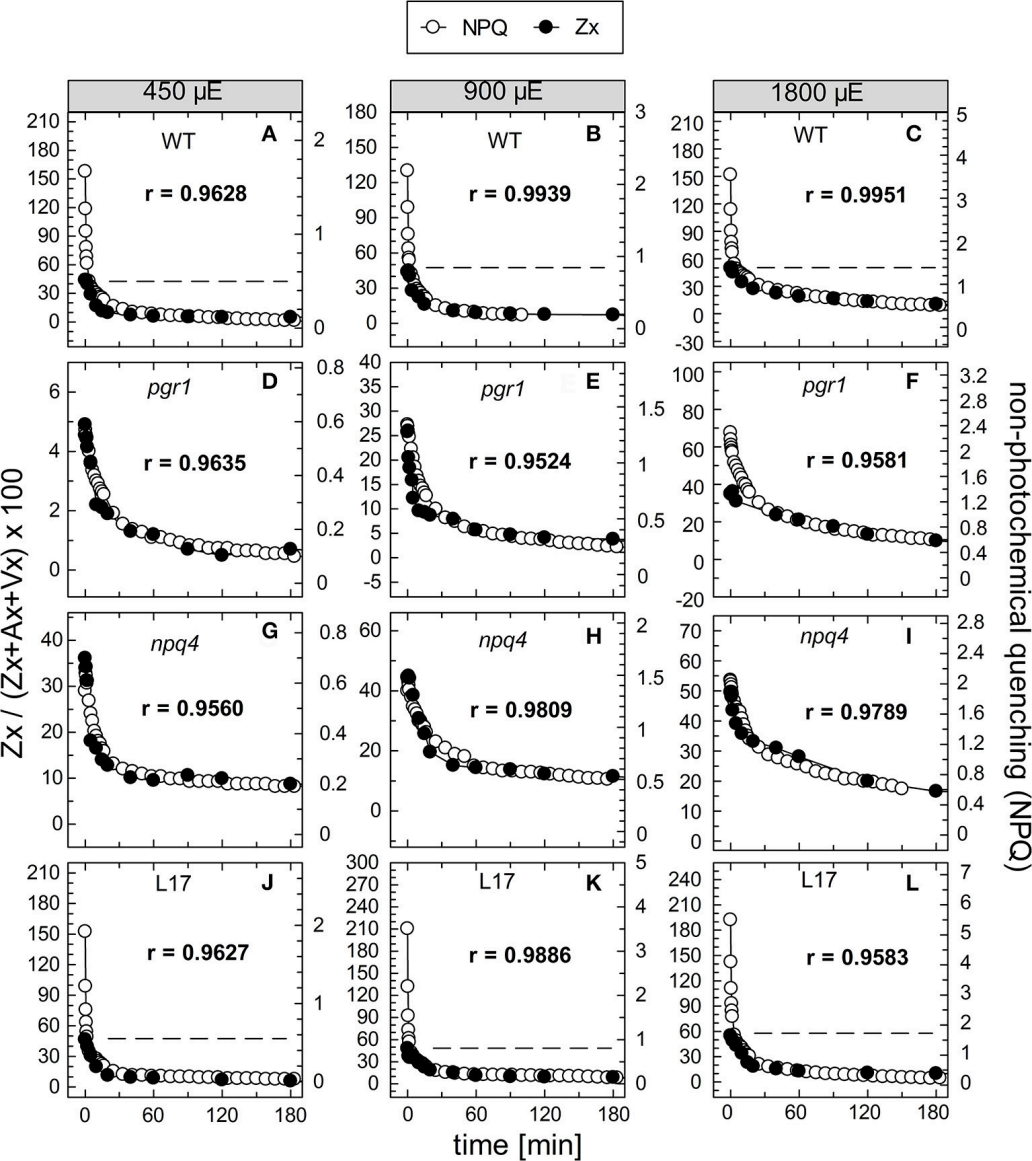
Comparison of the kinetics of NPQ relaxation and Zx epoxidation after 90 min of pre-illumination. The data for NPQ relaxation (open circles) and Zx epoxidation (filled circles) after 90 min of pre-illumination at the three actinic light intensities of 450, 900, and 1,800 μE are compared for the four genotypes with an active xanthophyll cycle: **(A–C)** WT, **(D–F)** *pgr1*, **(G–I)** *npq4* and **(J–L)** *L17*. For direct comparison, the data for Zx epoxidation were fitted to match the amplitudes of the slowly relaxing (> 2 min) NPQ components, only. The data were taken from Figure 6 (NPQ) and Figure 7 (Zx). The dotted lines in panels **(A–C)** and **(J–L)** indicate the NPQ amplitudes after relaxation of qE. The determined Pearson's correlation coefficient r is indicated in each panel.

To compare the kinetics of NPQ relaxation and Zx epoxidation, both data sets were plotted together for each time or pre-illumination and for all AL intensities upon normalization to the same initial amplitudes. This comparison is shown exemplarily for the data obtained after 90 min of pre-illumination in Figure 8, while the data for the three other conditions (15, 30, and 180 min of pre-illumination) are summarized in Figures S4–S6, respectively. Again, the respective Pearson's correlations coefficients derived from separate plots of NPQ vs Zx, are indicated in each panel. It is clearly visible, that the epoxidation of Zx occurred with slower kinetics than NPQ relaxation in the two qE-active genotypes, WT and *L17* (Figures 8A-C,J-L, respectively), and that particularly the rapid relaxation of the pH-dependent qE component of NPQ was not paralleled by Zx epoxidation (correlation coefficients between about 0.8 and 0.9). In contrast, Zx epoxidation in the qE-deficient *npq4* mutant followed quite similar kinetics than NPQ relaxation (Figures 8G–I) and thus yielded high correlation coefficients of about 0.98. In *pgr1* plants, however, Zx epoxidation kinetics matched NPQ relaxation kinetics after pre-illumination at the lowest AL intensity of 450 μE (Figure 8D), while Zx reconversion was more retarded than NPQ relaxation at increasing AL intensities (Figures 8E,F). The same general trends were observed for other times of pre-illumination (Figures S4–S6). These analyses indicate that Zx epoxidation is kinetically correlated with the pH-independent, slowly relaxing NPQ components, which have been assigned in WT plants to qZ and qI (Nilkens et al., 2010). For a better visualization of this correlation, the amplitudes of Zx epoxidation data were fitted to match the amplitudes of the more slowly relaxing NPQ components, qZ and qI, only. This analysis is again shown exemplarily for the data determined after 90 min of pre-illumination (Figure 9), while the data for 5, 30, and 180 min of pre-illumination are given in Figures S7–S9, respectively. When applying this way of analysis, the epoxidation of Zx matched nearly perfectly the kinetics of the slower, pH-independent NPQ components in the two qE-active genotypes, WT (Figures 9A–C) and *L17* (Figures 9J–L), as reflected by the high correlation coefficients. Note, that the dashed lines in the panels for these two genotypes indicate the NPQ amplitudes after relaxation of qE. In contrast, Zx epoxidation and NPQ relaxation were closely correlated for the entire time frame of the measurements in *npq4* mutants at all three AL intensities (Figure 9G–I), and for the two lowest AL intensities also in *pgr1* plants (Figures 9D,E). In the latter genotype, the correlation was restricted to the slowest phase of NPQ relaxation only at the highest AL intensity of 1,800 μE (Figure 9F). The same characteristics were observable for all other times of pre-illumination (Figures S7–S9). In conclusion, these analyses provide strong evidence that the kinetics of Zx epoxidation are closely correlated with the kinetics of slowly relaxing NPQ components in all genotypes with an active xanthophyll cycle.

## Discussion

The close kinetic correlation of Zx conversion and the dynamics of slowly inducible/relaxing NPQ states (qZ and qI) indicate an essential photoprotective function of Zx in these processes. While such a function has been proposed earlier, it remains a matter of debate, whether Zx is directly involved in the underlying quenching processes or whether it serves indirect functions as allosteric modulator of quenching processes or general photoprotective functions in the lipid phase of the membrane. Though the simple kinetical correlation cannot provide an answer to this question, the observed NPQ characteristics in different mutants support the view, that the slowly developing NPQ states can be induced independent of xanthophyll conversion. Such an interpretation provides a strong argument against a direct role of Zx in qZ and qI. This hypothesis is discussed in the following in context with the current knowledge of the literature related to a direct or indirect function of Zx in NPQ. It should further be noted, that the slowly inducible NPQ states are not related to state transitions (qT) or chloroplast movement. The contribution of qT to NPQ under these experimental conditions can be excluded from earlier work with the qT-deficient *stn7* mutant, which showed similar NPQ dynamics than WT plants under the same conditions (Nilkens et al., 2010). The possible contribution of chloroplast movement, which is triggered by blue light, was excluded from the comparison of the NPQ induction by either white (= blue and red light) or red actinic light (Figure S10). In both cases, very similar NPQ induction dynamics were determined.

### Quenching mechanisms and quenching sites

Any direct function of Zx in NPQ requires a specific binding site for the deactivation of excited Chl molecules. Because xanthophylls bind to Chl a/b antenna proteins only, direct quenching by Zx can be expected to occur exclusively in antenna proteins, but not in reaction center proteins. So far, a direct function of Zx has been proposed only for the pH-regulated qE mechanism. Experimental evidence for a direct Zx function, related to a charge transfer state, which involves electron transfer from Zx to Chl, was derived from transient absorption measurements in the near infrared region (Holt et al., 2005; Ahn et al., 2008; Avenson et al., 2008, 2009; Dall'Osto et al., 2017). This mechanism was described to be active in isolated thylakoid membranes (Holt et al., 2005) and in isolated minor PSII antenna complexes Lhcb4-6 (Ahn et al., 2008; Avenson et al., 2008), but not in trimeric LHCII (Avenson et al., 2008). From recent analysis of the subunit and Chl organization in the PSII C_2_S_2_M_2_ supercomplex from Arabidopsis it was concluded, that Lhcb5 (CP26), Lhcb4 (CP29), and LHCII S-trimers can transfer excitation energy directly to the reaction center core (CP43), while LHCII M-trimers transfer excitation energy either through S-trimers or Lhcb4 (CP29) (van Bezouwen et al., 2017). This suggests that energy flow from the antenna to the reaction center occurs mainly through S-trimers or Lhcb4, but not through Lhcb5, which makes Lhcb5 a much less favorable candidate for efficient quenching compared to Lhcb4. Recent analysis of mutants devoid of minor antenna complexes provided evidence that two mechanisms of qE quenching exist: one in minor antenna complexes, which involves the formation of a xanthophyll cation radical and another in trimeric LHCII, which does not involve a charge transfer state but requires Zx (Dall'Osto et al., 2017). Although it remains to be clarified, to what extent these characterized charge transfer states contribute to qE under *in vivo* conditions in the fully assembled PSII antenna, these studies indicate a dual role of Zx in energy dissipation, combining a direct and indirect function.

In contrast, an exclusively indirect function of Zx in qE is favored by Horton, Ruban and co-workers, who suggest that Zx mainly modulates the efficiency of qE by shifting the pH-dependence of qE to higher pH values (Horton et al., 2000). In their model, energy dissipation in antenna proteins is facilitated by energy transfer to lutein (Ruban et al., 2007), and activation of quenching is essentially triggered by pH-regulated conformational changes in PSII antenna proteins (for a review see Ruban et al., 2012). Finally, a carotenoid independent quenching mechanism based on the formation of a Chl-Chl charge transfer state has been postulated as mechanism for qE quenching on basis of Chl fluorescence and transient absorption spectroscopy (Miloslavina et al., 2008; Muller et al., 2010). Based on simulations of the molecular dynamics of monomeric PSII antenna proteins, it has recently been proposed that Zx rather supports conformational changes at the luminal side of antenna proteins without direct contribution to NPQ (Papadatos et al., 2017).

Hence, all different approaches support an indirect role of Zx in qE, while an additional direct function was proposed on basis of transient absorption spectroscopy. It should be noted, however, that all of these studies are limited by the fact that no intact leaves have been used as material. This is an important issue, since NPQ activation is a complex property of the entire chloroplast, and activation of qE involves not only structural reorganization of PSII complexes but also of the thylakoid membrane (Kirchhoff, 2014; Demmig-Adams et al., 2015; Schumann et al., 2017). The structural properties and NPQ characteristics of isolated thylakoid membranes and recombinant antenna proteins can be expected to differ from that of intact chloroplasts. Applying time-resolved fluorescence analysis to intact, detached Arabidopsis leaves, Holzwarth and co-workers identified two different quenching sites that were activated under *in vivo* conditions along with NPQ induction (Holzwarth et al., 2009). One site was found to depend on the presence of Zx and was localized to antenna proteins associated with PSII reactions center. It remained unclear, however, whether Zx acts as direct quencher of energy or simply as regulator of a Zx-independent quenching process and whether the Zx-dependent quenching site contributes to qE or to qZ.

For the two slowly inducible and relaxing NPQ components, qZ and qI, no detailed mechanisms have been proposed so far. For qZ, the quenching has been assigned to processes involving the minor antenna protein Lhcb5 (Dall'Osto et al., 2005), but it remains to be elucidated, whether also other antenna proteins contribute to qZ. As pointed out above, however, analysis of the energy transfer pathways in PSII-LHCII supercomplexes do not favor energy quenching in Lhcb5 (van Bezouwen et al., 2017). Moreover, the exact role of Zx in qZ is still unclear. The induction of NPQ states with similar characteristics as qZ in the two xanthophyll cycle mutants *npq1* and *npq2* (Figures 1, 6) argue against any direct function of Zx in qZ. The photoinhibitory quenching qI is based on the high-light induced damage of the reaction center protein D1, but the underlying molecular quenching mechanism is unknown. A direct role Zx in qI seems very unlikely, since Zx binding to PSII reaction center proteins has not been observed. The close correlation of the relaxation of qZ and qI with Zx epoxidation thus strongly supports an indirect role Zx in qZ and qI.

### Zx binding sites and Xanthophyll conversion characteristics

A direct function of Zx in energy dissipation involving a Chl-Zx charge transfer state has been assigned to Zx located at the L2 site (Holt et al., 2005; Ahn et al., 2008; Avenson et al., 2008), while quenching by lutein has been proposed to occur at the L1 site (Ruban et al., 2007; Avenson et al., 2009). Thus, a direct function of Zx essentially requires binding to the L2 site in minor antenna proteins. The light-induced exchange of Vx by Zx at the L2 site is therefore a prerequisite of a direct quenching function of Zx. Binding of Vx to the L2 site has been reported for Lhcb6 (Passarini et al., 2009) and Lhcb4 (Pan et al., 2011; Wei et al., 2016). In Lhcb5, this binding site is predominantly occupied by Lut (Wei et al., 2016), but may also be occupied by Vx (Ballottari et al., 2009). It is under debate, however, to what extent Vx bound at the L2 site is convertible to Zx and whether the formed Zx rebinds to the L2 site. It is known from *in vitro* studies that de-epoxidation of Vx bound to the L2 site of the minor complexes Lhcb4-6 occurs to a rather low extent (about 10–40%) only, and follows slower kinetics than at the V1 site of trimeric LHCII proteins (Morosinotto et al., 2003; Wehner et al., 2004). Consequently, only a fraction of minor complexes can be expected to bind Zx upon activation of qE and qZ. It is further questionable, whether the formed Zx indeed rebinds to the L2 site. Recent comparative analysis of antenna complexes isolated from plants in the NPQ active and NPQ inactive state did not provide any evidence for Zx binding to L2 (Xu et al., 2015), which argues against an exchange of Vx by Zx at the L2 site. In conclusion, binding of Zx to the minor antenna complexes Lhcb4-6 at L2 is rather unlikely and occurs, if at all, only to very low extent. This view is further supported by the general characteristics of xanthophyll conversion. The de-epoxidation of Vx to Zx occurs in the lipid phase of the thylakoid membrane (Jahns et al., 2009) and does not require a specific interaction of the lumen-localized VxDE, which catalyzes this reaction, with antenna proteins. The latter has been derived from the notion that Vx conversion in isolated Arabidopsis thylakoids can be catalyzed from both sides of the membrane with similar kinetics and efficiency (Macko et al., 2002). Based on this work, it was postulated that the release and diffusion of Vx in the lipid phase is the rate-limiting step of Vx conversion. Interestingly, conversion of Vx to Zx from the stroma side has recently been shown to be active under *in vivo* conditions in the green alga *Chlamydomonas reinhardtii* (Li et al., 2016). In this case, the Vx converting enzyme is exclusively located in the chloroplast stroma, supporting the view that diffusion of Vx in the lipid phase precedes its conversion. Conversion of Vx in the lipid phase requires initially the release of Vx from the respective binding site, explaining why Vx bound to the peripheral V1 site occurs very rapidly and with high efficiency (Jahns et al., 2001). The release of Vx from the inner L2 site in minor PSII antenna proteins thus likely requires a pronounced structural reorganization, and the same applies to any specific rebinding of the formed Zx to the L2 site. The latter is complicated by the different properties of Vx and Zx related to structure and hydrophobicity. This makes it much more likely that Zx acts either in the membrane or at the periphery of antenna complexes rather than being rebound at very specific inner xanthophyll binding sites like L2.

### The role of non-protein bound Zx

A general function of Zx in photoprotection independent of its role in energy dissipation has been derived from studies of different Arabidopsis mutants (Havaux and Niyogi, 1999; Havaux et al., 2007; Dall'Osto et al., 2010). Such a function is likely related to the antioxidative capacity of Zx as non-protein bound xanthophyll in the lipid phase of the membrane (Havaux et al., 2007; Dall'Osto et al., 2010). The amount of the xanthophyll cycle pigments (VAZ pool size) as well as the portion of photoconvertible Vx is known to increase upon acclimation to high light intensities in parallel with an increased NPQ capacity (Adams and Demmig-Adams, 1992; Demmig-Adams and Adams, 1992; Bailey et al., 2004; Demmig-Adams et al., 2006; Schumann et al., 2017). Because the number of antenna proteins and thus xanthophyll binding sites is reduced upon high light acclimation, the additional VAZ pigments must reside in the lipid phase of the membrane. This underlines the important role of non-protein bound Zx in photoprotection upon long-term high light stress. It is further known that the loss of Zx correlates with increased levels of tocopherols and *vice versa* (Havaux et al., 2000, 2005; Triantaphylides and Havaux, 2009), suggesting a central role of Zx in the protection against lipid peroxidation. Moreover, non-protein bound Zx may serve in the regulation of membrane properties such as fluidity or stability (Tardy and Havaux, 1997; Havaux, 1998). The close correlation of Zx levels and photoinhibitory quenching found here supports the earlier observation that ZEP activity is down-regulated concomitant with PSII activity under prolonged high light stress (Reinhold et al., 2008) and might thus reflect the necessity to retain Zx in the lipid phase for photoprotection of inactivated PSII.

Up to 3 h illumination at the highest light intensity of 1,800 μE used here, no pronounced impact of different Zx levels on the slowly inducible NPQ components (qZ and qI) was observable (Figures 1, 2). This implies that the inactivation of PSII occurs independent of the presence of Zx, suggesting a photoprotective role of Zx in longer lasting high light stress, as has been shown for the Zx-deficient *npq1* mutant (Havaux and Niyogi, 1999; Havaux et al., 2000). The gradual down-regulation of Zx epoxidation in parallel with the recovery from photoinhibition thus rather reflects a protective role of Zx during PSII repair cycle than a direct function of Zx in qI quenching. The coordinated down-regulation of both Zx epoxidation and NPQ relaxation upon increasing high-light stress requires a strict co-regulation of both processes. So far, the molecular mechanism of the down-regulation of ZEP activity is unknown. However, recent work showed that mutants with deficient NADPH thioredoxin reductase C (NTRC) accumulate higher levels of Zx, indicating a partial inactivation of ZEP in absence of thioredoxin reduction (Naranjo et al., 2016). This supports a crucial role of thioredoxin mediated reduction of disulphides in the regulation of ZEP activity and suggests that oxidation of cysteine residues might be responsible for ZEP inactivation under prolonged high light stress. The retainment of high Zx levels along with the repair of damaged PSII seems to be central for protecting PSII centers during the repair cycle. Related to such a role, the Zx content and the Zx dynamics thus represent a molecular memory of experienced high light stress. From the physiological point of view, this will allow not only photoprotection of inhibited PSII centers, but likely also a more rapid or efficient reactivation of NPQ states after recurrent high light stress.

In conclusion, the comparison of the dynamics of NPQ and Zx levels strongly support the hypothesis that Zx has no direct function in the quenching mechanisms related to the two slowly relaxing NPQ components qZ and qI. This interpretation is derived from the similar characteristics of these two NPQ components independent of the different Zx levels in the studied genotypes. The observed slight differences in the NPQ development in the different mutants as compared to WT plants might be either due to differences in the qE properties or might reflect alterations in the modulation of PSII down-regulation in dependence of Zx, as has been shown also for qE. Hence, Zx has likely an allosteric function in the modulation of slowly inducible NPQ states, which might additionally involve general photoprotective functions in the lipid phase of the membrane.

## Author contributions

PJ: Planned and designed the experiments; EK: Performed the experiments; Both authors analyzed and interpreted the data. PJ wrote the MS. Both authors read and approved the final version of the manuscript.

### Conflict of interest statement

The authors declare that the research was conducted in the absence of any commercial or financial relationships that could be construed as a potential conflict of interest.
