# Supplementary material for: The Dynamics of Energy Dissipation and Xanthophyll Conversion in Arabidopsis Indicate an Indirect Photoprotective Role of Zeaxanthin in Slowly Inducible and Relaxing Components of Non-photochemical Quenching of Excitation Energy
Source: Front Plant Sci. 2017 Dec 8;8:2094. doi: 10.3389/fpls.2017.02094 (PMC5727089; doi:10.3389/fpls.2017.02094)
Supplement: Supplementary file 1 [file SupplementaryTables.pdf]

Supplementary Material

The Dynamics of Energy Dissipation and Xanthophyll Conversion in Arabidopsis Indicate an Indirect Photoprotective Role of Zeaxanthin in Slowly Inducible and Relaxing Components of Non-photochemical Quenching of Excitation Energy

Eugen Kress, Peter Jahns*

*** Correspondence:** Peter Jahns: pjahns@hhu.de

Table S1 Kinetic parameters of NPQ dynamics upon illumination at 450 µE. The data of NPQ induction (Ind) and relaxation after different time of pre-illumination shown in Figure 1 and Figure 2, respectively, were fitted with two exponentials according to the equation
$y = A_1*\exp(-t/\tau_1) + A_2*\exp(-t/\tau_2) + y_0$.

	time	A_1	τ_1[s]	A_2	τ_2 [s]	y_0
WT	Ind	1.35	54			
	5	1.25	28	0.26	658	0.00
	30	0.99	48	0.39	716	0.00
	90	1.00	79	0.37	1090	0.06
	180	1.01	76	0.34	1650	0.13
pgr1	Ind	0.21	98	0.43	2830	
	5	0.21	1090	0.02	3820	0.00
	30	0.28	750	0.21	> 5000	0.00
	90	0.32	830	0.17	> 5000	0.08
	180	0.39	620	0.23	3920	0.08
L17	Ind	2.65	75			
	5	2.06	19	0.37	370	0.00
	30	1.78	25	0.39	630	0.04
	90	1.49	37	0.48	850	0.05
	180	1.53	33	0.53	450	0.08
npq4	Ind	0.27	157	0.39	3350	
	5	0.26	540	0.05	2440	0.00
	30	0.32	540	0.25	> 5000	0.00
	90	0.37	580	0.11	> 5000	0.17
	180	0.37	460	0.24	> 5000	0.11
npq2	Ind	1.21	12			
	5	1.28	81	0.15	700	0.00
	30	0.98	70	0.31	810	0.00
	90	1.31	117	0.31	1400	0.04
	180	1.13	95	0.31	> 5000	0.02
npq1	Ind	0.53	23			
	5	0.28	13	0.27	550	0.01
	30	0.28	320	0.14	1420	0.07
	90	0.42	180	0.42	2470	0.04
	180	0.34	75	0.35	3080	0.14

Table S2 Kinetic parameters of NPQ dynamics upon illumination at 900 µE. The data of NPQ induction (Ind) and relaxation after different time of pre-illumination shown in Figure 1 and Figure 2, respectively, were fitted with two exponentials according to the equation

$y = A_1*\exp(-t/\tau_1) + A_2*\exp(-t/\tau_2) + y_0$.

	time	A_1	τ_1 [s]	A_2	τ_2 [s]	y_0
WT	Ind	1.49	49	0.81	790	
	5	1.41	29	0.34	710	0.01
	30	1.57	36	0.56	1060	0.09
	90	1.42	37	0.68	800	0.21
	180	1.38	56	0.56	1790	0.32
pgr1	Ind	0.15	30	1.02	1240	
	5	0.24	520	0.13	2840	0.02
	30	0.29	430	0.42	2240	0.20
	90	0.74	646	0.41	> 5000	0.19
	180	0.65	667	0.34	> 5000	0.17
L17	Ind	3.18	89	0.48	1070	
	5	2.72	27	0.54	295	0.02
	30	2.80	31	0.82	780	0.06
	90	2.67	32	0.67	1090	0.14
	180	2.86	58	0.84	1840	0.13
npq4	Ind	0.37	150	0.82	2765	
	5	0.33	465	0.08	3870	0.04
	30	0.52	640	0.17	3410	0.16
	90	0.39	1030	0.45	> 5000	0.40
	180	0.47	590	0.50	> 5000	0.27
npq2	Ind	1.45	20	0.63	1609	
	5	1.50	110	0.15	1210	0.05
	30	1.45	103	0.45	1520	0.04
	90	1.40	97	0.49	1250	0.22
	180	1.39	135	0.64	2170	0.26
npq1	Ind	0.54	11	0.84	1630	
	5	0.32	11	0.36	640	0.04
	30	0.38	33	0.61	1170	0.24
	90	0.49	360	0.47	3200	0.25
	180	0.52	260	0.48	4200	0.40

Table S3 Kinetic parameters of NPQ dynamics upon illumination at 1800 µE. The data of NPQ induction (Ind) and relaxation after different time of pre-illumination shown in Figure 1 and Figure 2, respectively, were fitted with two exponentials according to the equation

$y = A_1*\exp(-t/\tau_1) + A_2*\exp(-t/\tau_2) + y_0$.

	time	A_1	τ_1[s]	A_2	τ_2[s]	y_0
WT	Ind	1.54	30	2.12	857	
	5	1.69	41	0.28	895	0.1
	30	2.30	64	0.91	1920	0.27
	90	2.07	47	0.93	2630	0.52
	180	2.12	57	0.97	2200	0.47
pgr1	Ind	0.13	10	2.76	1372	
	5	0.57	620	0.12	> 5000	0.07
	30	0.79	144	1.03	2180	0.37
	90	0.80	184	1.18	2620	0.58
	180	0.93	147	1.31	6040	0.65
L17	Ind	1.73	35	3.67	690	
	5	2.07	11	1.20	228	0.14
	30	3.91	44	1.02	1060	0.18
	90	3.61	47	1.52	1240	0.31
	180	3.25	54	1.22	1961	0.73
npq4	Ind	0.23	41	1.78	1215	
	5	0.36	212	0.38	2600	0.08
	30	0.53	405	0.79	3920	0.47
	90	0.84	720	1.07	> 5000	0.11
	180	0.33	260	1.46	4600	0.63
npq2	Ind	1.59	14	1.49	1449	
	5	1.76	133	0.26	1830	0.03
	30	1.87	70	0.64	1560	0.08
	90	1.68	130	1.20	3180	0.28
	180	1.63	194	1.09	> 5000	0.51
npq1	Ind	0.61	8	1.83	1185	
	5	0.29	44	0.46	1170	0.08
	30	0.41	140	1.30	1820	0.64
	90	0.59	185	0.77	2200	0.81
	180	0.70	295	0.88	2600	0.66
