# Supplementary material for: The Dynamics of Energy Dissipation and Xanthophyll Conversion in Arabidopsis Indicate an Indirect Photoprotective Role of Zeaxanthin in Slowly Inducible and Relaxing Components of Non-photochemical Quenching of Excitation Energy
Source: Front Plant Sci. 2017 Dec 8;8:2094. doi: 10.3389/fpls.2017.02094 (PMC5727089; doi:10.3389/fpls.2017.02094)

Supplementary Material

The Dynamics of Energy Dissipation and Xanthophyll Conversion in Arabidopsis Indicate an Indirect Photoprotective Role of Zeaxanthin in Slowly Inducible and Relaxing Components of Non-photochemical Quenching of Excitation Energy

Eugen Kress, Peter Jahns*

*** Correspondence:** Peter Jahns: pjahns@hhu.de

Figure S1 NPQ dynamics. The induction of NPQ during 180 min of illumination at three different actinic light intensities (450 µE, 900 µE and 1800 µE of white light), and the relaxation of NPQ after pre-illumination for 5 min, 30 min, 90 min and 180 min at each actinic light intensity were determined for **(A-C)** WT, **(D-F)** *pgr1*, **(G-I)** *L17*, **(J-L)** *npq4*, **(M-O)**, *npq2* and **(P-R)** *npq1* plants. During the whole measurements, detached leaves were placed on wet paper in a temperature-controlled cuvette (20°C) under permanent supply with ambient air. Mean values of 3-6 independent measurements are shown.

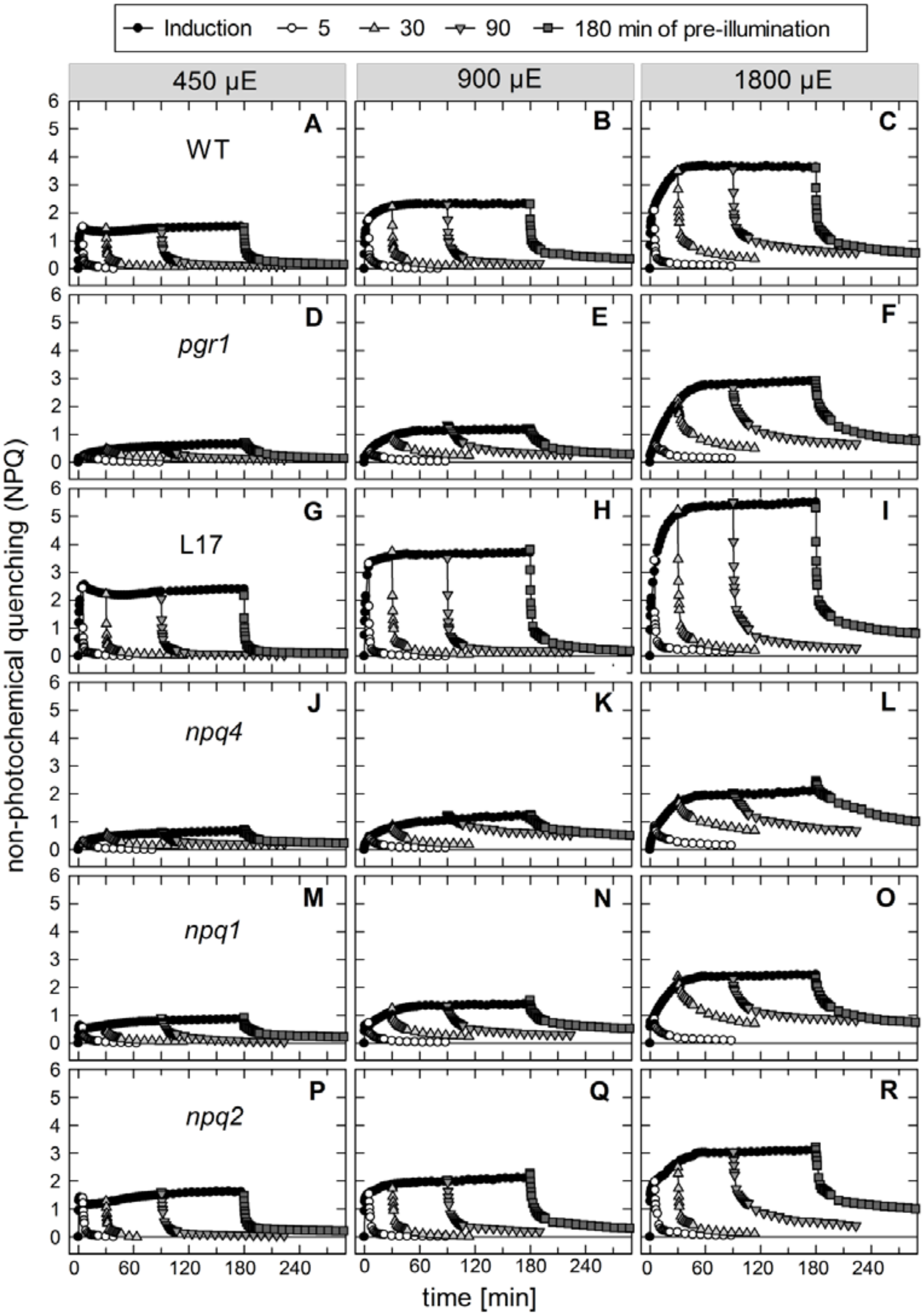

Figure S2 Zx dynamics The synthesis of Zx during 180 min of illumination at three different actinic light intensities (450 µE, 900 µE and 1800 µE of white light), and the epoxidation of Zx after different times of pre-illumination at each actinic light intensity were determined for **(A-C)** WT, **(D-F)** *pgr1*, **(G-I)** *L17*, **(J-L)** *npq4*. For *pgr1* plants, Zx epoxidation is shown only for the longer pre-illumination times of 90 and 180 min, because only very low amounts of Zx were present at the shorter pre-illumination times of 5 and 30 min. During the whole measurements, detached leaves were placed on wet paper in a temperature-controlled cuvette (20°C) under permanent supply with ambient air. Mean values of 3-6 independent measurements +/- SE are shown.

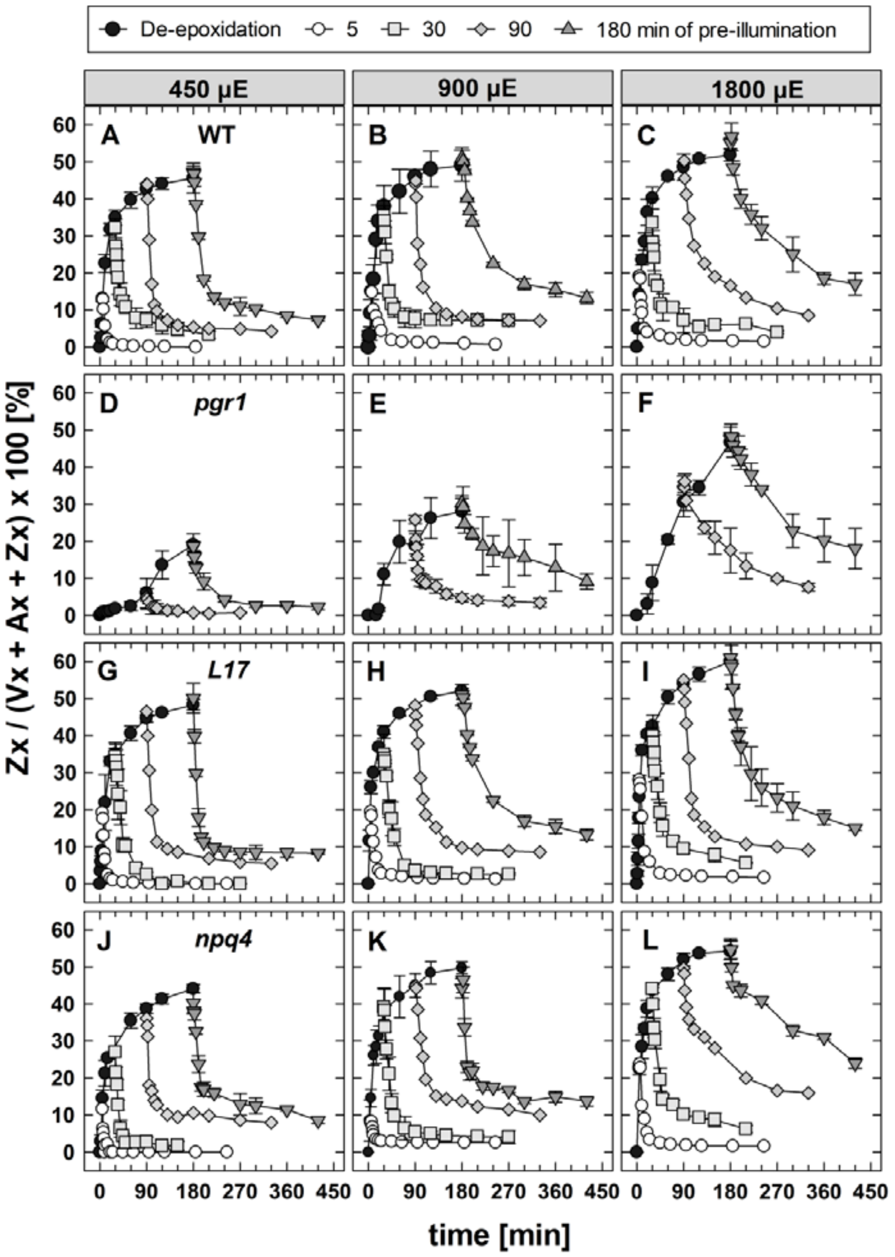

Figure S3 Correlation analysis of NPQ and Zx dynamics. (A, B) NPQ induction and Zx synthesis. **(C, D)** NPQ relaxation and Zx epoxidation. The data show the results from the experiment with WT plants upon illumination at 900 µE. The data for NPQ induction and Zx synthesis **(A, B)** were taken from Figure 4B, the NPQ relaxtion and Zx epoxidation **(C, D)** was analyzed after pre-illumination for 90 min (Figure 8B). The left side **(A, C)** shows the data for the total NPQ amplitude, while the data related to the qE component were omitted on the right side **(B, D).** The determined Pearson's correlation coefficient r is indicated in each panel.

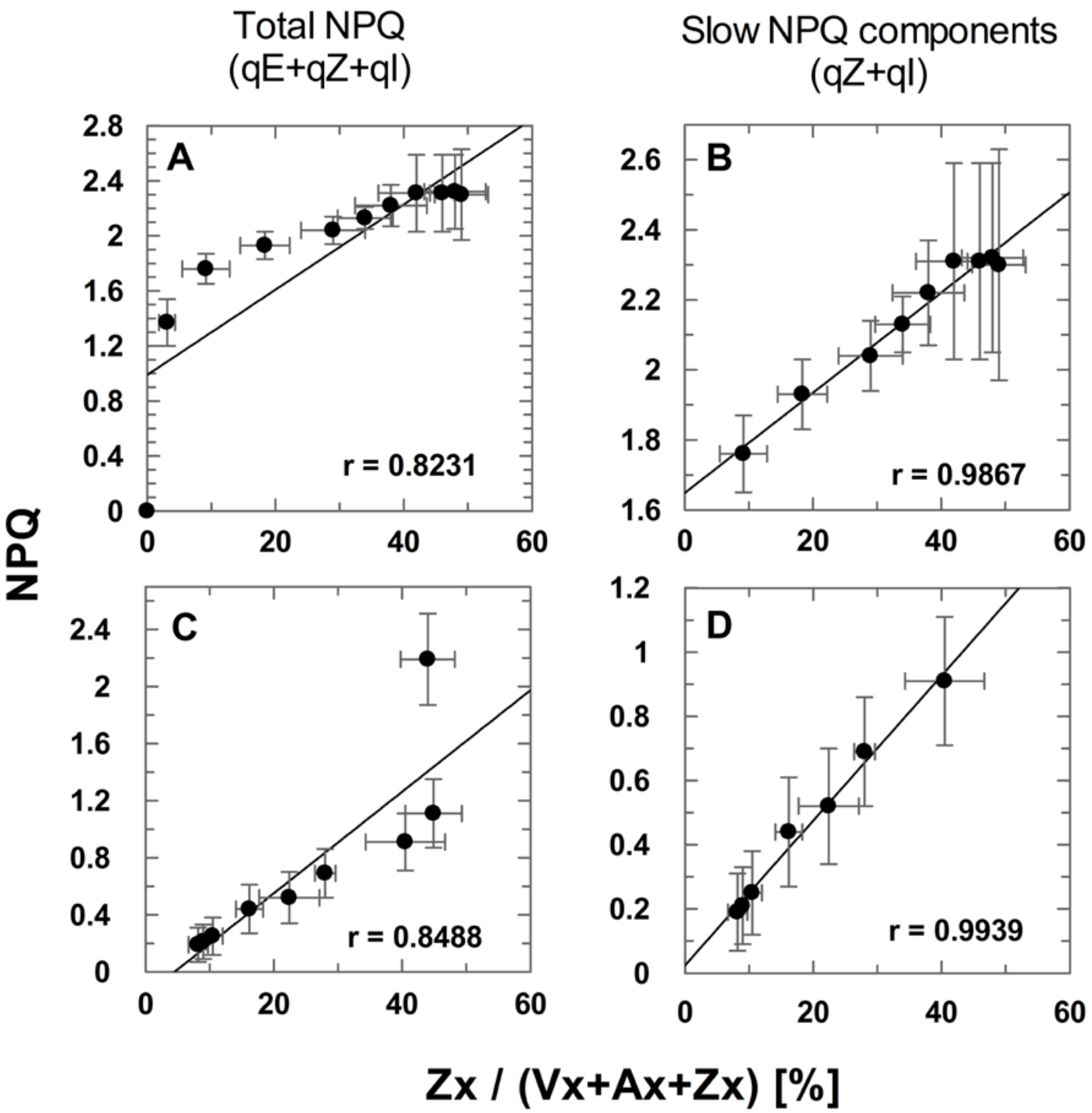

Figure S4 Comparison of NPQ relaxation and Zx epoxidation after 5 min of pre-illumination. The time course of NPQ relaxation and Zx epoxidation after 5 min of pre-illumination at the three actinic light intensities of 450 µE, 900 µE and 1800 µE is compared for: **(A-C)** WT, **(D-F)** *npq4*, and **(G-I)** *L17*. No data for *pgr1* plants were included, because only very low amounts of Zx were present after 5 min of pre-illumination. The data were taken from Figure 6 (NPQ) and Figure 7 (Zx). The determined Pearson's correlation coefficient r is indicated in each panel.

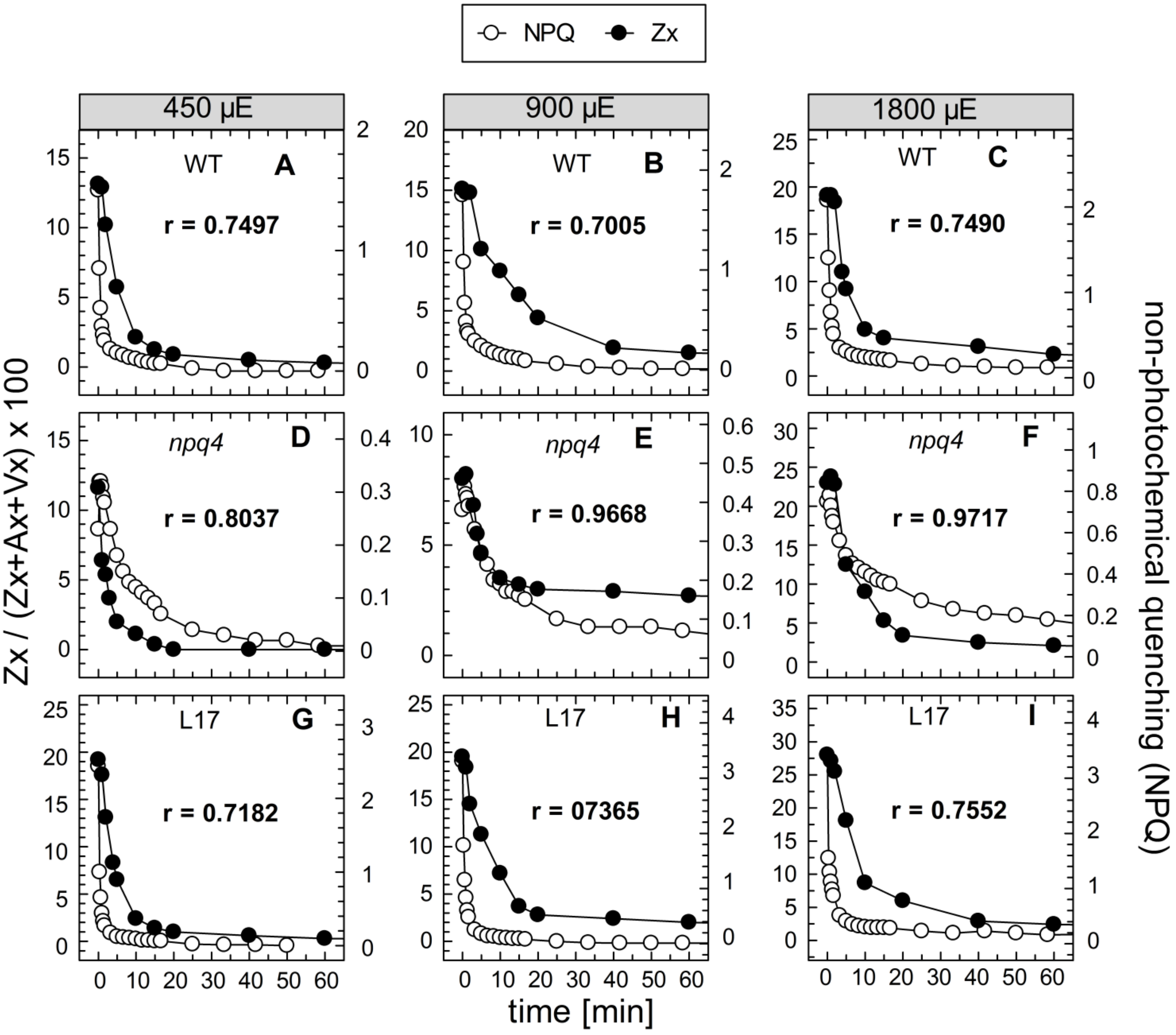

Figure S5 Comparison of NPQ relaxation and Zx epoxidation after 30 min of pre-illumination. The time course of NPQ relaxation and Zx epoxidation after 30 min of pre-illumination at the three actinic light intensities of 450 µE, 900 µE and 1800 µE is compared for: **(A-C)** WT, **(D-F)** *npq4*, and **(G-I)** *L17*. No data for *pgr1* plants were included, because only low amounts of Zx were present after 30 min of pre-illumination. The data were taken from Figure 6 (NPQ) and Figure 7 (Zx). The determined Pearson's correlation coefficient r is indicated in each panel.

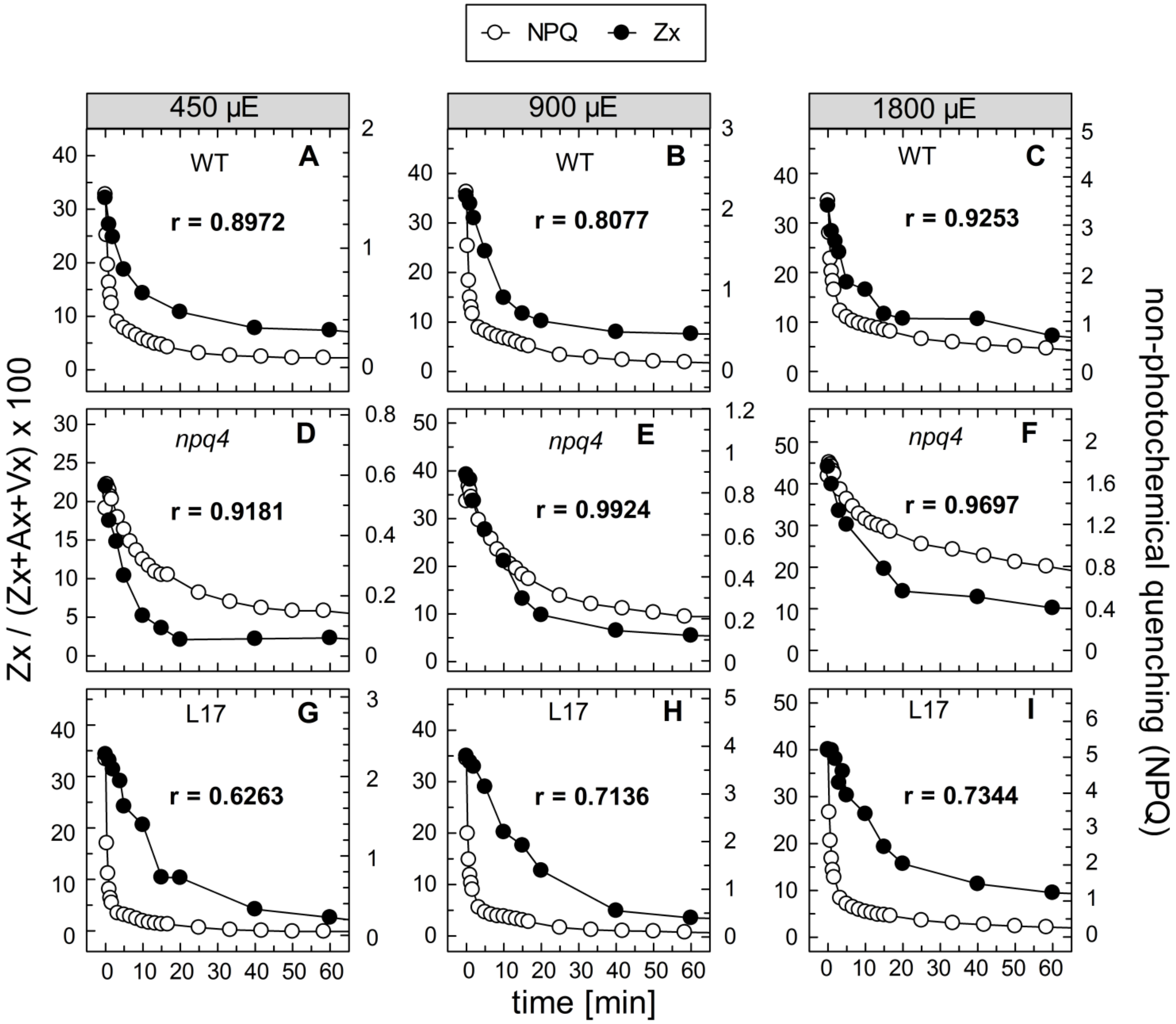

Figure S6 Comparison of NPQ relaxation and Zx epoxidation after 180 min of pre-illumination. The time course of NPQ relaxation and Zx epoxidation after 180 min of pre-illumination at the three actinic light intensities of 450 µE, 900 µE and 1800 µE is compared for the four genotypes with an active xanthophyll cycle: **(A-C)** WT, **(D-F)** *pgr1*, **(G-I)** *npq4* and **(J-L)** *L17*. The data were taken from Figure 6 (NPQ) and Figure 7 (Zx). The determined Pearson's correlation coefficient r is indicated in each panel.

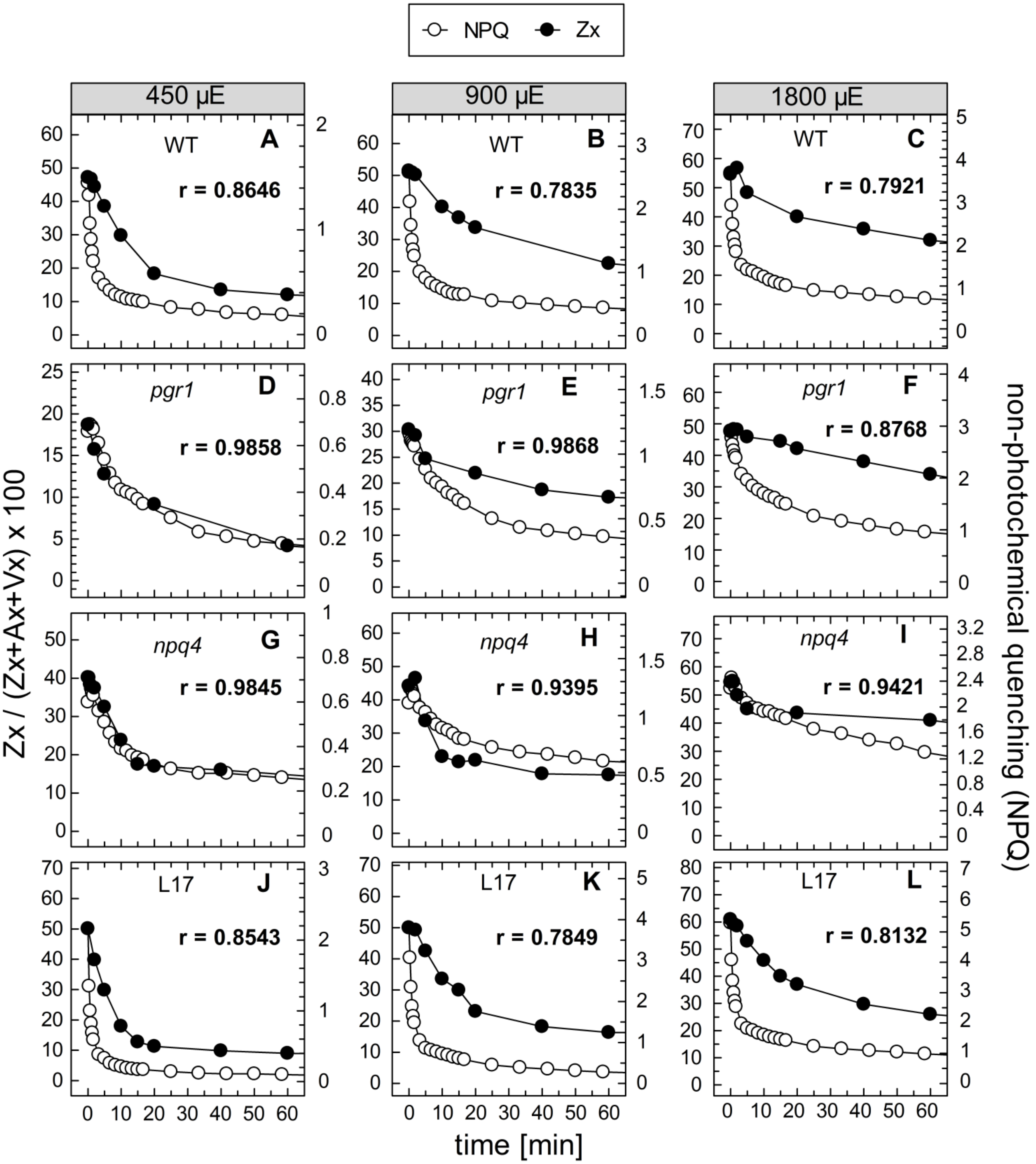

Figure S7 Comparison of the kinetics of NPQ relaxation and Zx epoxidation after 5 min of pre-illumination. The data for NPQ relaxation (open circles) and Zx epoxidation (filled circles) after 5 min of pre-illumination at the three actinic light intensities of 450 µE, 900 µE and 1800 µE are compared for: **(A-C)** WT, **(D-F)** *pgr1*, **(G-I)** *npq4* and **(J-L)** *L17*. **(A-C)** WT, **(D-F)** *npq4*, and **(G-I)** *L17*. No data for *pgr1* plants were included, because only very low amounts of Zx were present after 30 min of pre-illumination. For direct comparison, the data for Zx epoxidation were fitted to match the amplitudes of the slowly relaxing (> 2 min) NPQ components, only. The data were taken from Figure 6 (NPQ) and Figure 7 (Zx). The dashed lines in panels A-C and G-I indicate the NPQ amplitudes after relaxation of qE. The determined Pearson's correlation coefficient r is indicated in each panel.

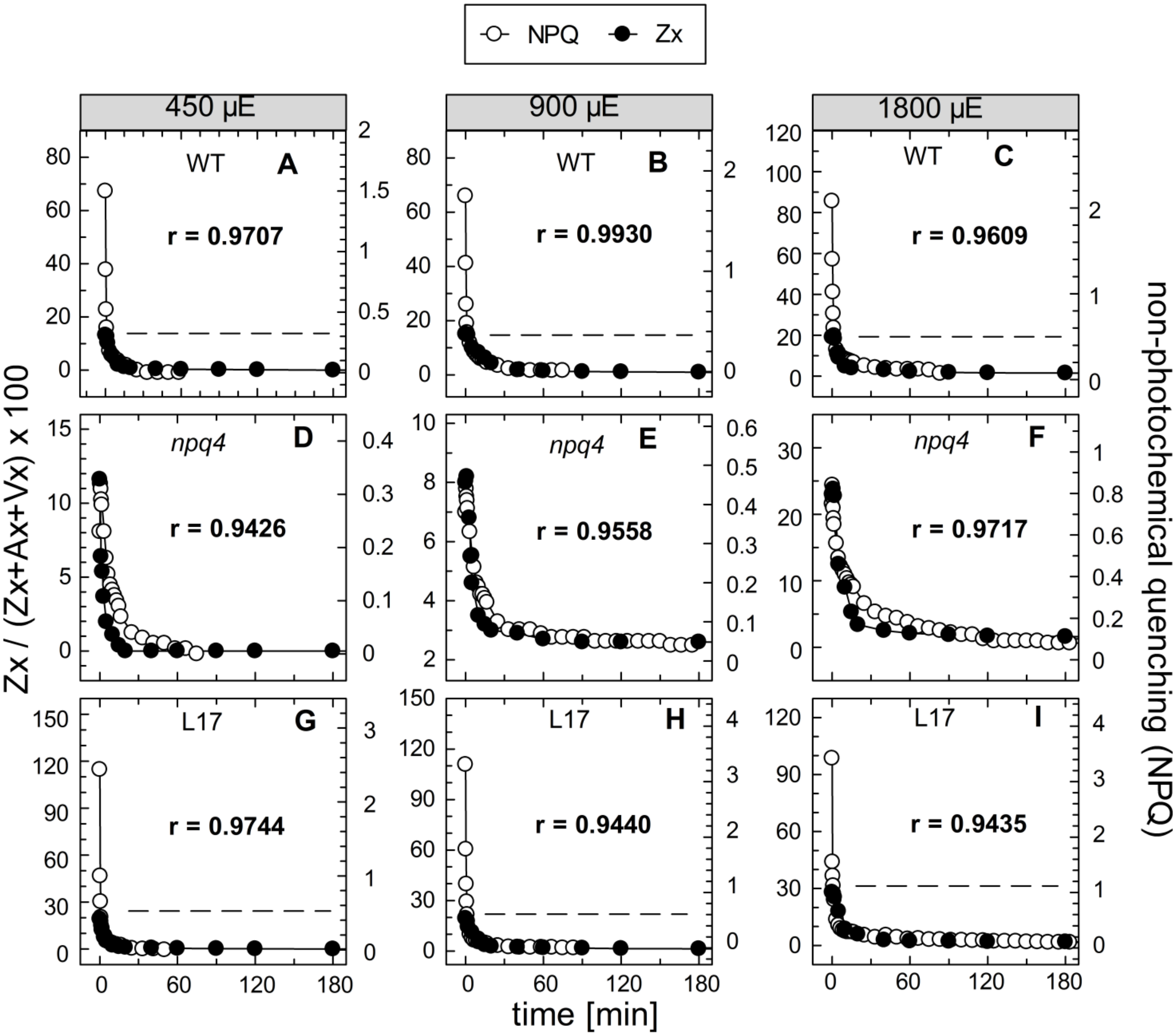

Figure S8 Comparison of the kinetics of NPQ relaxation and Zx epoxidation after 30 min of pre-illumination. The data for NPQ relaxation (open circles) and Zx epoxidation (filled circles) after 30 min of pre-illumination at the three actinic light intensities of 450 µE, 900 µE and 1800 µE are compared for: **(A-C)** WT, **(D-F)** *pgr1*, **(G-I)** *npq4* and **(J-L)** *L17*. **(A-C)** WT, **(D-F)** *npq4*, and **(G-I)** *L17*. No data for *pgr1* plants were included, because only low amounts of Zx were present after 30 min of pre-illumination. For direct comparison, the data for Zx epoxidation were fitted to match the amplitudes of the slowly relaxing (> 2 min) NPQ components, only. The data were taken from Figure 6 (NPQ) and Figure 7 (Zx). The dashed lines in panels A-C and G-I indicate the NPQ amplitudes after relaxation of qE. The determined Pearson's correlation coefficient r is indicated in each panel.

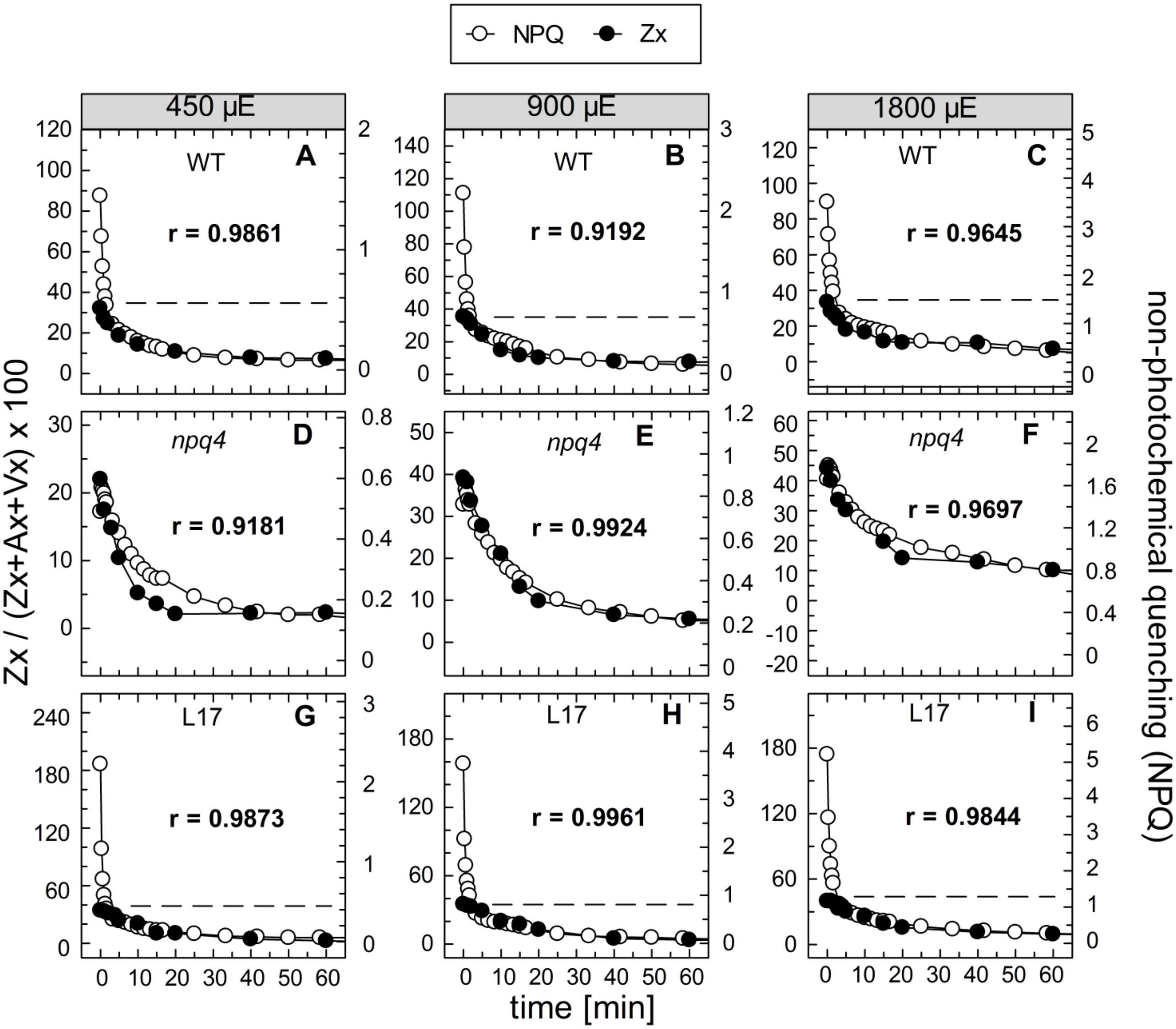

Figure S9 Comparison of the kinetics of NPQ relaxation and Zx epoxidation after 180 min of pre-illumination. The data for NPQ relaxation (open circles) and Zx epoxidation (filled circles) after 180 min of pre-illumination at the three actinic light intensities of 450 µE, 900 µE and 1800 µE are compared for the four genotypes with an active xanthophyll cycle: **(A-C)** WT, **(D-F)** *pgr1*, **(G-I)** *npq4* and **(J-L)** *L17*. For direct comparison, the data for Zx epoxidation were fitted to match the amplitudes of the slowly relaxing (> 2 min) NPQ components, only. The data were taken from Figure 6 (NPQ) and Figure 7 (Zx). The dashed lines in panels A-C and J-L indicate the NPQ amplitudes after relaxation of qE. The determined Pearson's correlation coefficient r is indicated in each panel.

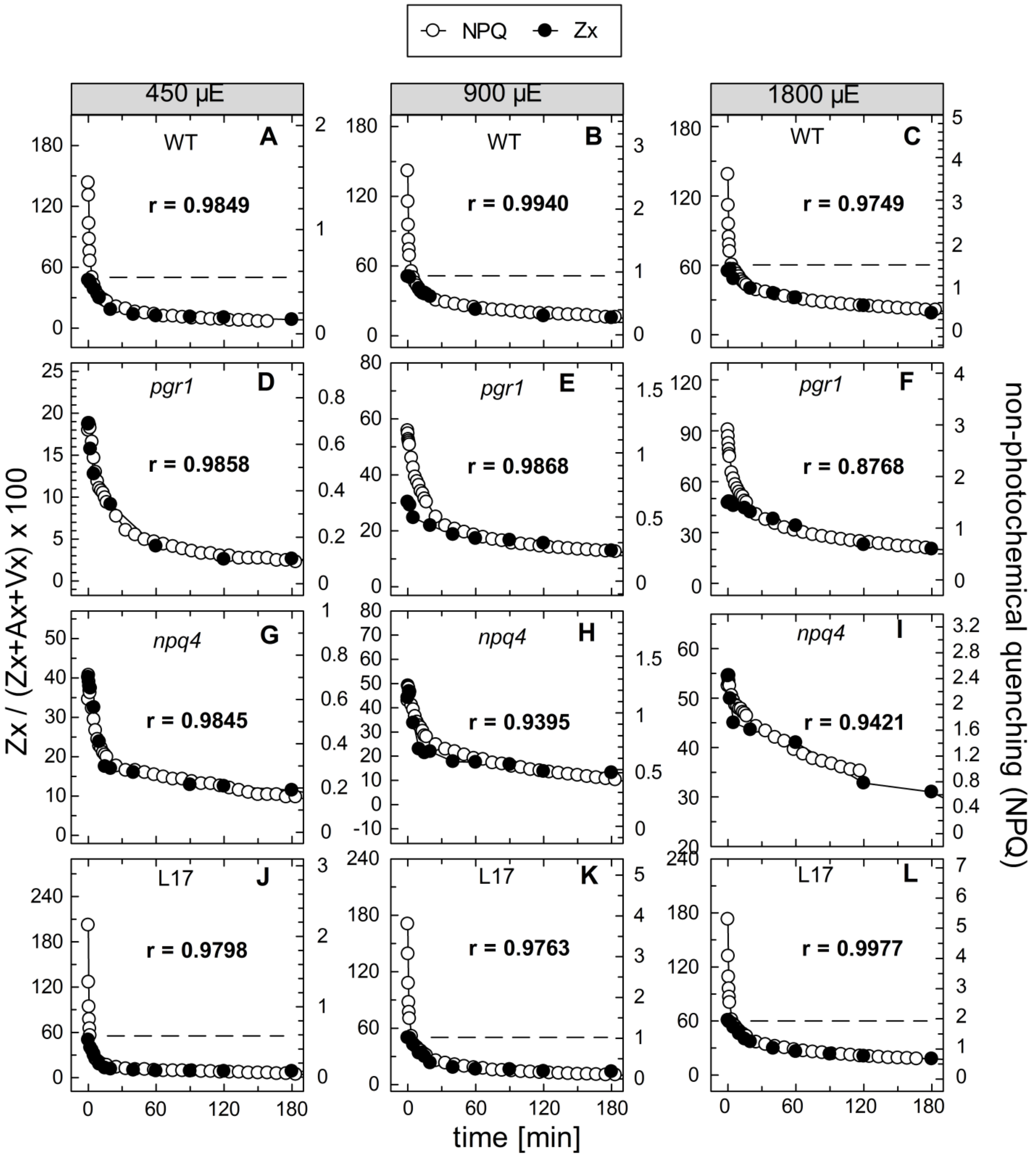

Figure S10 Comparison of NPQ induction upon illumination with white or red actinic light. The data for NPQ induction with white light (WL, white symbols) were taken from Figure 1. NPQ induction with red light (620 nm, RL, red symbols) was measured with the DUAL PAM 100 (Walz, Effeltrich, Germany). Actinic light intensities are indicated in the Figure. Note, that about 20% lower intensities were used for RL. The data represent mean values (± SD) of 3-5 independent measurements.

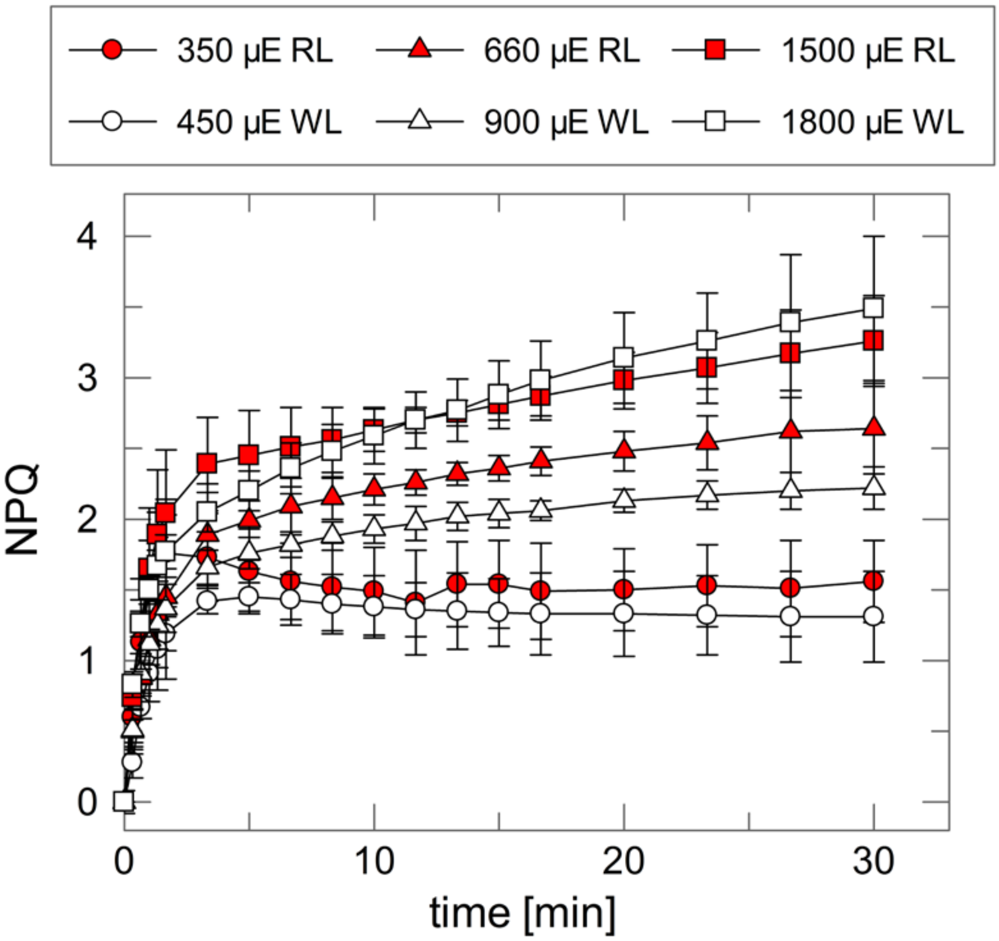

Supplement: Supplementary file 2 [file SupplementaryFigures.pdf]
